# Auditable unit-aware thresholds in symbolic regression via logistic-gated operators

**DOI:** 10.1016/j.isci.2026.117092

**Published:** 2026-08-06

**Authors:** Ou Deng, Ruichen Cong, Jianting Xu, Shoji Nishimura, Atsushi Ogihara, Qun Jin

**Affiliations:** 1 Graduate School of Human Sciences, Waseda University, Tokorozawa, Saitama 359-1192, Japan; 2 Faculty of Human Sciences, Waseda University, Tokorozawa, Saitama 359-1192, Japan

**Keywords:** symbolic regression, genetic programming, logistic-gated operators, unit-aware thresholds, model auditability, interpretable machine learning, cut-point discovery

## Abstract

Symbolic regression offers the promise of readable equations but often struggles to represent unit-aware thresholds that drive clinical decisions. We propose logistic-gated operators (LGOs)—differentiable gates with learnable location and steepness—embedded as typed primitives in a genetic-programming SR engine. Training is performed in standardized space; thresholds are mapped back to physical units for audit. On three public health datasets (MIMIC-IV ICU, eICU, NHANES), LGO_hard_ identifies cut-points that appear clinically plausible: 4/13 fall within 10% of guideline anchors, 7/13 within 20%; the remaining gates tend to shift toward extreme-risk or early-warning regimes. On the derived high-risk classification tasks (based on score thresholds), compared with AutoScore, LGO achieves comparable or modestly improved area under the receiver operating characteristic curve (AUROC) and calibration while providing explicit thresholds rather than point tables. On smooth UCI benchmarks, gates are frequently pruned, helping to preserve parsimony. The approach yields relatively compact symbolic equations with auditable, unit-aware thresholds that may support clinical decision-making.

## Highlights

• Unit-aware thresholds as learnable primitives in symbolic expressions
• LGO matches AutoScore AUROC while providing explicit gate structure
• Hard gates concentrate on clinical variables; soft fallback when smooth
• Reproducible pipeline with threshold-audit tooling and Wilcoxon tests

## Graphical abstract

**Figure:**
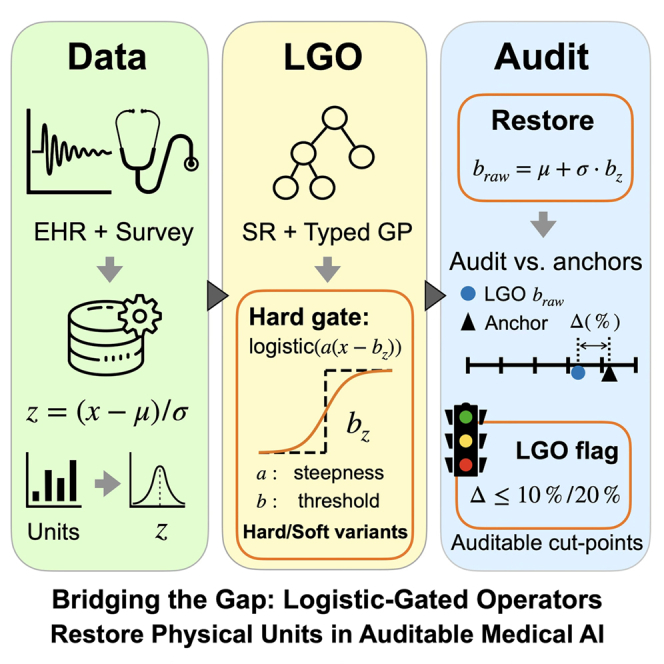

## Teaser

Health sciences; Machine learning

## Introduction

### Motivation and background

Symbolic regression (SR) searches over analytic expressions that can summarize patterns in data and, in some settings, make modeling assumptions more explicit.^1^^,^^2^ Compared with black-box predictors, SR may yield compact formulas whose structure and parameters are directly inspectable, which can support scientific interpretation and regulatory traceability when deployed in high-stakes settings.^3^^,^^4^ In medicine and public health, interpretability often entails more than a readable expression: clinicians and regulators may need to know where a model changes its behavior, in what physical units, and how those cut-points compare to domain anchors or guidelines.^5^^,^^6^ Examples include mean arterial pressure (MAP) targets in sepsis care,^7^^,^^8^ blood-pressure tiers for cardiovascular risk management,^9^ lipid and metabolic cut-points,^10^^,^^11^ and glucose thresholds used for screening or diagnosis.^12^ Governance guidance similarly emphasizes auditable, well-calibrated models with transparent update pathways.^13^

Two data ecosystems make these requirements concrete. First, critical-care electronic health record (EHR) cohorts (e.g., MIMIC-IV ICU and the multi-center eICU database) enable outcome modeling from routinely measured physiologic signals.^14^^,^^15^^,^^16^^,^^17^ Many of these variables—MAP, lactate, respiratory rate, vasopressor use, neurologic status—are interpreted relative to operational or guideline thresholds, so recovered turning points are most useful when they can be stated and checked in natural units. Second, population surveys such as the National Health and Nutrition Examination Survey (NHANES) provide nationally representative measurements of cardiometabolic risk factors where anchors (e.g., systolic blood pressure [BP] tiers, high-density lipoprotein [HDL] minima, waist-circumference bands, fasting-glucose ranges) are embedded in screening programs.^18^^,^^19^ In both settings, beyond accuracy, stakeholders may value models that introduce as few switches as necessary and present those switches explicitly for clinical review.^20^

Classical SR typically represents thresholded behavior indirectly by composing arithmetic and smooth primitives. While flexible, this can yield long expressions with implicit cut-points that are difficult to locate and audit. Existing interpretable systems, such as point-based clinical scores (e.g., AutoScore^21^^,^^22^) and generalized additive models with per-feature shape functions (e.g., explainable boosting machines (EBMs)^23^), expose human-readable structure but do not treat thresholds as first-class, unit-aware parameters inside a free-form equation. Motivated by this gap, we propose a family of LGOs: symbolic primitives that encode gating with learnable location and steepness in standardized space and map those parameters back to physical units for audit. Our empirical focus is deliberately health-centric (ICU, eICU, and NHANES); rather than positioning LGO as a single-number state-of-the-art predictor, we emphasize executable interpretability—equations whose thresholds can be reported in natural units and reviewed against domain anchors.^3^^,^^4^

### Related work and limitations

#### Symbolic regression families and search heuristics

SR originated in genetic programming (GP), which evolves expression trees over primitive sets.^1^^,^^24^^,^^25^ Discoveries of compact laws and invariants have suggested SR’s potential for scientific applications.^2^ Subsequent work broadened search and selection, from strong typing and semantic constraints to selection schemes tailored for regression.^26^^,^^27^ Contemporary surveys benchmark diverse engines and highlight the trade-off space between fit and parsimony.^28^ Production-grade toolchains increasingly standardize operators and fitness reporting (e.g., Operon, PS-Tree, PySR, RILS-ROLS).^29^^,^^30^^,^^31^^,^^32^ Recent extensions to the SR paradigm also illustrate the field’s evolution: boosting frameworks seek to improve symbolic regressors by stacking a small number of high-capacity stages^33^; exhaustive search enumerates all expressions up to a specified complexity and ranks them via minimum description length to provide optimality guarantees^34^; multi-objective evolutionary schemes penalize expression size to alleviate overfitting and yield Pareto-optimal trade-offs between accuracy and sparsity^35^; and counterexample-driven genetic programming incorporates formal constraints to help ensure domain-specific properties while learning interpretable expressions.^36^ These developments explore orthogonal ways of balancing accuracy, complexity, and interpretability within the SR landscape, and may offer complementary perspectives to the approach presented here.

#### Hybrid, differentiable, and neuro-symbolic approaches

Beyond evolutionary search, hybrid SR injects gradients, priors, or neural scaffolds with the aim of improving data efficiency and extrapolation. Physics-inspired pipelines and differentiable surrogates exemplify this direction.^37^^,^^38^ Deep SR and neural-symbolic integrations further leverage policy gradients and representation learning.^39^^,^^40^ Program-structure priors and grammar models help guide the hypothesis space,^41^^,^^42^ while differentiable architecture search can influence operator choice.^43^ Recent work also applies neural-guided symbolic regression to discovering governing equations of networked dynamical systems: ND^2^ reduces high-dimensional network searches to equivalent one-dimensional systems via dedicated network operators and uses a pre-trained neural model to guide symbolic search.^44^ While this setting differs from our focus on auditable unit-aware cut-points, it further illustrates the potential value of combining learned representations with symbolic discovery. These systems typically focus on optimizing expressions but generally do not return unit-aware thresholds as first-class parameters within the final symbolic model.

#### Gating and piecewise mechanisms

Decades of neural modeling have highlighted the utility of gates for regime switching (e.g., LSTM/GRU) and specialization (mixture-of-experts).^45^^,^^46^^,^^47^ Theoretical analyses of partitioning capacity (e.g., linear-region counts) provide insight into how gated compositions may encode piecewise structure.^48^ LGO draws on this heritage but seeks to incorporate gates into symbolic expressions whose parameters are trained in *Z* score space and then inverted to natural units, with the aim of enabling direct audit against anchors.

#### Interpretable learners, clinical scores, and post-hoc explanations

Rule lists, soft decision trees, and inherently interpretable models offer explicit conditional or additive structure.^49^^,^^50^^,^^51^ In clinical prediction, point-based scoring systems such as AutoScore provide sparse, integer-valued scores with simple decision rules,^21^^,^^22^ while generalized additive models with shape constraints—e.g., the EBMs implemented in InterpretML^23^—learn smooth per-feature curves that can be visualized and edited. Model-agnostic toolkits add local/global attributions and partial effects on top of black-box models. Integer-programming-based scoring systems such as RiskSLIM^52^ construct integer-coefficient risk scores but require proprietary MIP solvers and scale only to a handful of features; scoring-table evolutionary tuning (SET) refines existing scoring tables via multi-objective evolutionary optimization. However, these approaches generally either do not produce free-form analytic formulas interleaving gates with algebra, or they treat thresholds as implicit artifacts of the score table or the explainer (e.g., bin boundaries, knots). LGO aims to make thresholds part of the expression tree—with parameters audited in natural units—to facilitate numerical comparison against clinical or engineering anchors. In our experiments, we attempt to benchmark this approach directly against AutoScore- and EBM-style baselines.

#### Scientific machine learning and operator learning

Symbolic discovery connects naturally to physics-informed and operator-learning frameworks that encode structure and units.^53^^,^^54^ Neural operator families and universal-approximation results have highlighted the utility of parameterized kernels with semantic meaning (e.g., periods, diffusion scales).^55^^,^^56^^,^^57^ Domain-equivariant architectures further illustrate how inductive bias can be aligned with scientific symmetries.^58^ LGO is motivated by a similar philosophy, but applied to thresholds: the parameters (*a*, *b*) are assigned explicit roles (steepness and location) and are mapped back to physical units for audit.

#### Health data, anchors, and governance

We evaluate on public cohorts with documented variable definitions (MIMIC-IV ICU, eICU, NHANES)^14^^,^^15^^,^^16^^,^^17^^,^^18^ and anchor sets reflecting guideline practice: sepsis hemodynamic targets,^7^^,^^8^ blood pressure and lipid management,^9^^,^^10^ metabolic syndrome criteria,^11^ and glucose classification standards.^12^ Such alignment may facilitate clinical review and regulatory dialogue.^6^^,^^13^ For non-clinical benchmarks (cardiotocography [CTG], Cleveland, Hydraulic), we reference UCI repositories for precise feature definitions.^59^^,^^60^^,^^61^ Beyond clinical guidelines, many engineering domains also have established threshold phenomena that could potentially serve as external anchors for auditing learned cut-points—for example, percolation thresholds in electrically conducting ceramic composites.^62^

#### Evaluation practice and sensitivity

Our protocol reports mean ± std across seeds and audits thresholds via natural-unit inversion, accompanied by sanity checks (root mean squared error [RMSE] ≥ mean absolute error [MAE]; internal-external metric consistency). We use variance-based sensitivity tools when appropriate to probe stability.^63^^,^^64^ For causal interpretability and data issues (e.g., confounding, missingness), we draw on established literatures to inform audit and robustness practices.^65^^,^^66^^,^^67^

#### Where LGO fits—and its limits

Compared with arithmetic-only SR,^1^^,^^28^ neuro-symbolic pipelines,^37^^,^^38^ and established interpretable learners such as AutoScore and EBM,^21^^,^^22^^,^^23^ LGO attempts to provide unit-aware, auditable gates as primitives. The hard-gate variant tends to yield sparser switching than a soft multiplicative gate, which may be better suited to our target use-cases in ICU, eICU, and NHANES. In our experiments, LGO achieved discrimination broadly comparable to AutoScore while producing expressions that are considerably less complex than high-capacity ensembles such as EBM. Limitations persist: on globally smooth relations (e.g., Hydraulic), gates may over-parametrize; anchors must be curated carefully (counts/categorical codes or age-dependent “normals” can mislead); and budgets for learning (*a*, *b*) matter. These boundaries argue for a mechanism-aware choice of primitives and for reporting both accuracy and parsimony.^3^ Emerging SR directions—concept libraries, interactive co-design, and robustness studies—offer complementary perspectives and may benefit from making thresholds first-class, auditable objects.^68^^,^^69^^,^^70^^,^^71^^,^^72^

### Our proposal: Logistic-gated operator (LGO) family

To help address the expressivity-auditability gap, we propose enriching the SR primitive set with an LGO that aims to bring smooth gates and explicit thresholds into the symbolic search itself. We refer to this primitive as an LGO. Throughout, “unit-aware” denotes physical measurement units (mmHg, mmol/L, mg/dL), not the notion of unit as in “operator.” We adopt two canonical forms:

(Equation 1) $${LGO}_{\text{soft}}\left(x;a,b\right)=x\;\sigma \left(a\left(x-b\right)\right),\;\sigma \left(z\right)=\frac{1}{1+e^{-z}},$$

(Equation 2) $${LGO}_{\text{hard}}\left(x;a,b\right)=\sigma \left(a\left(x-b\right)\right).$$

Here b∈R encodes a threshold and *a* > 0 controls transition steepness. Equation 1 preserves magnitude while gating (“soft” gate); Equation 2 isolates a probabilistic gate (“hard” tendency). LGOs are closed under composition: replacing *x* with a subexpression *f*(*x*) yields expression-level gating $f\left(x\right)\;\sigma \left(a\left(f\left(x\right)-b\right)\right)$; multi-input gates arise via products/sums of sigmoids, e.g., AND_2_(*x*, *y*) = *σ*(*a*(*x* − *b*)) *σ*(*a*(*y* − *b*)) and ${OR}_{2}\left(x,y\right)=1-\left(1-\sigma \left(a\left(x-b\right)\right)\right)\left(1-\sigma \left(a\left(y-b\right)\right)\right)$.

We integrate LGOs into a strongly typed SR search to separate feature inputs (Feat), positive steepness (Pos), and thresholds (Thr), with the goal of constraining the hypothesis space and facilitating targeted mutations on (*a*, *b*).^1^^,^^25^^,^^26^

### Theoretical properties and inductive bias

#### Heaviside limit and calibration

For fixed *b*, $\sigma \left(a\left(x-b\right)\right)\to 1_{\left\{x>b\right\}}$ pointwise as *a* → *∞*; on any compact set avoiding *x* = *b*, the convergence is uniform. Hence LGO_*hard*_ approaches a step and LGO_*soft*_ approaches *x* **1**_{*x*>*b*}_. The parameter *a* therefore calibrates gate sharpness from graded modulation to near-discrete switching.

#### Smoothness, gradients, and regularization

Both gates are differentiable for finite *a*; for Equation 1,

$$\frac{\partial }{\partial b}\;{LGO}_{soft}\left(x;a,b\right)=-a\;x\;\sigma \left(a\Delta \right)\left(1-\sigma \left(a\Delta \right)\right),\;\frac{\partial }{\partial a}\;{LGO}_{soft}\left(x;a,b\right)=x\;\Delta \;\sigma \left(a\Delta \right)\left(1-\sigma \left(a\Delta \right)\right),$$

where Δ = *x* − *b*. The gating factor has global Lipschitz constant ≤ *a*/4 in *x*, which spreads transitions unless the data support sharp changes. This yields an implicit regularization bias toward smooth regime boundaries, tightened as *a* grows. The full operator list and properties are described in Section S4 of supplemental information.

#### Approximation of piecewise structure

Sigmoids approximate indicators and thus arbitrary continuous functions via superpositions.^73^^,^^74^ Combining this with multiplicative masking suggests a constructive approximation for piecewise-*C*^1^ maps: given *k* distinct thresholds on one dimension, there exists an LGO expression with *O*(*k*) sigmoid factors whose uniform error on compact subsets (excluding the discontinuity loci) can be made arbitrarily small. In multiple dimensions, products of sigmoids approximate indicators of axis-aligned polytopes, allowing for smooth partitioning of the domain into regimes that may then be modeled by simple algebraic subexpressions. In this sense, LGOs aim to provide SR with a more direct means of expressing threshold-based behavior, rather than relying on lengthy arithmetic surrogates.^2^^,^^37^

#### Typed semantics and search economy

Typing (Feat,Pos,Thr) restricts where each symbol may appear and confines (*a*, *b*) to their appropriate domains. This is intended to prune invalid trees, improve search efficiency, and support micro-mutations focused on (*a*, *b*) while preserving global structure.^26^^,^^27^ In turn, the hard gate may induce a bias toward parsimonious switching (fewer active gates), while the soft gate tends to favor graded modulation—an inductive bias we examine empirically on ICU and NHANES.

#### Why coordinate descent?

**Figure 1:**
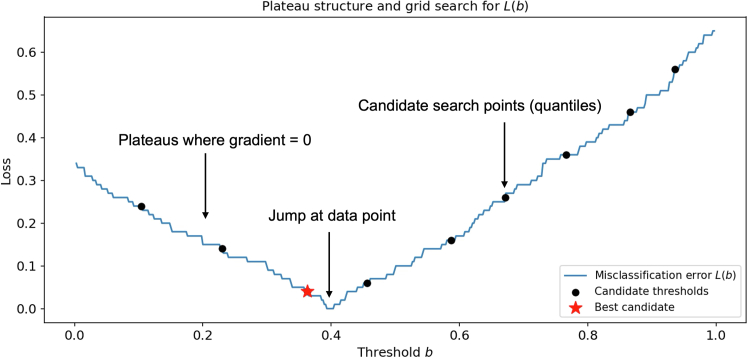
Plateau loss landscape and grid search for *L*(*b*) For a toy dataset, the misclassification loss *L*(*b*) of a hard-gated model displays a stepped landscape as the threshold *b* varies: the loss remains constant (forming plateaus) when *b* moves between adjacent data points and drops abruptly when *b* crosses a sample (a cliff). Standard candidate search or direct search illustration stalls on these plateaus because the gradients vanish, whereas local coordinate descent is able to jump across them by directly evaluating the loss at a discrete set of candidate thresholds (black dots) and selecting the optimal one (red star). Implementation uses Gaussian perturbations; see SI S5

In LGO, we optimize not only the symbolic structure but also the continuous gate parameters (*a*, *b*) that scale and shift the logistic gating function. When the steepness *a* is large, the logistic gate approximates a hard step, and the resulting loss landscape *L*(*b*) in the threshold *b* consists of wide plateaus separated by steep drops. In such a landscape gradient-based optimizers tend to perform poorly: the derivative $\sigma \left(a\left(x-b\right)\right)\left[1-\sigma \left(a\left(x-b\right)\right)\right]$ almost vanishes on the plateaus and becomes extremely large in the narrow transition regions, so updates may either stall or diverge. To address this, we fix the expression structure and apply a local coordinate descent on (*a*, *b*). Holding all other parameters constant, we perturb *b* and select an improved value, thereby attempting to leap across the flat regions where gradients vanish. Alternating between updates for *a* and *b* tends to converge efficiently without relying on unstable gradient information. As illustrated in Figure 1, the misclassification loss for a hard-gated model is indeed piecewise constant between data points, and evaluating a handful of candidate thresholds (black dots) can help identify a good solution (red star). Concretely, for each gated feature, we apply a stochastic coordinate search that alternately perturbs the threshold *b* and the steepness *a*. In even-numbered steps, *b* is updated by adding Gaussian noise (*σ* = 0.25 in standardized units) to the current value; in odd-numbered steps, *a* is rescaled by a factor drawn from the candidate set {0.8, 1.2, 2.0, 3.0}. Each candidate is evaluated via 5-fold cross-validation on the training split and accepted only if it reduces the validation loss. The procedure iterates for up to *S* steps (150 in our experiments) with a patience window of 15 (details in the supplemental information).

### Unit-aware threshold recovery

Expressions are evolved in standardized (*Z* score) space for numerical stability and comparability across features. For a feature *x* with training statistics (*μ*_*x*_, *σ*_*x*_), a learned threshold ${\hat{b}}_{z}$ maps back to natural units via

$${\hat{b}}_{\text{raw}}=\mu_{x}+\sigma_{x}\;{\hat{b}}_{z}.$$

This unit-aware inversion is performed with train-only statistics to avoid leakage and enables direct audit against domain anchors (e.g., MAP 65 mmHg, lactate 2 mmol/L, SBP tiers, HDL minima, fasting-glucose ranges).^8^^,^^9^^,^^10^^,^^11^^,^^12^ For expression-level gates $f\left(x\right)\;\sigma \left(a\left(f\left(x\right)-b\right)\right)$, we estimate the *z*-standardization of *f*(*x*) on the training fold and apply the same inversion principle.

### Contributions

We introduce LGOs as typed, unit-aware gating primitives for symbolic regression and summarize their limiting behavior, smoothness, and approximation capacity. We develop a strongly typed, hybrid search that couples GP structure evolution with explicit optimization of the gate parameters (*a*, *b*), and we invert learned thresholds from standardized space to natural units using training-split statistics to avoid leakage and enable post-hoc audits against external anchors. In our health-cohort experiments, the hard-gate variant typically yields sparser switching structures while maintaining predictive performance broadly comparable to established SR baselines under matched evaluation budgets.

### Paper organization

Results first present fully specified exemplar equations, then report benchmark performance under matched budgets, followed by unit-aware threshold audits, parsimony comparisons, and ablations within the LGO family. Discussion considers auditability and governance, deployment considerations, case studies, and limitations. STAR Methods provide full technical details on the LGO-SR framework, datasets, experimental protocol, and statistical analysis.

## Results

### Interpretable exemplar equations and full model specification

To foreground model transparency, we select an exemplar LGO_hard_ model on each clinical dataset from the full top-*k* candidate pool (10 seeds × 10 candidates = 100 models per dataset), prioritizing clinical interpretability while requiring competitive accuracy. Table 1 summarizes each exemplar’s variables, active gates, key thresholds (in natural clinical units), and test *R*^2^. The seed, within-seed rank, and selection criteria are documented in supplemental information section S3.1 and Table S4.

**Table 1:** Exemplar LGO_hard_ models selected from the top-*k* candidate pool on each clinical dataset

Dataset	Variables	Active gates	Key thresholds	Test *R*^2^
ICU (MIMIC) — gate-free smooth model	lactate, creatinine,	none	–	0.846
	MAP, vasopressor	–	–	–
eICU — multi-organ gated model (6 gates)	GCS, resp. rate, mech. vent.,	GCS ≤12.3	lactate >3.4 mmol/L	0.808
	vasopressor, lactate,	resp. rate, mech. vent.,	GCS: reduced	–
	HR, creatinine	vasopressor, lactate,	consciousness	–
	–	HR + creatinine	–	–
NHANES — metabolic threshold model (2 active gates)	systolic BP, waist,	SBP ≥126 mmHg	SBP: Stage 1 hypertension	0.803
	fasting glucose,	glucose ≥98 mg/dL	glucose: impaired fasting glucose	–
	triglycerides, gender	–	–	–

Models were chosen from all seeds to illustrate the range of structures discovered by LGO—from gate-free arithmetic compositions (ICU) through multi-organ gated assessments (eICU). Seed, within-seed rank, and full expressions are reported in Table S4 for reproducibility.

#### Three modes of LGO

The three exemplars illustrate qualitatively distinct model structures, demonstrating that LGO adapts its use of gating to the statistical properties of each dataset.

On **ICU**, the exemplar (seed 21, rank 1, *R*^2^ = 0.846) is a gate-free smooth model:

(Equation 3) $${\hat{y}}_{\text{ICU}}\approx \sqrt{\;3\;\text{lactate}-\ln\left(\text{creat.}\right)-\ln\left(\text{lactate}\right)-4.35-\text{MAP}\;}+\text{vasopressor}.$$

Although the raw expression contains a creatinine gate (*a* = 7.37, *b*_std_ = 2.02), it is numerically inactive: the surrounding division by near-zero renders the gate-containing term negligible (see SI for the full expression). The four selected variables—lactate, creatinine, MAP and vasopressor use—cover four of the six Sequential Organ Failure Assessment (SOFA) organ dimensions, and the logarithmic and square-root transforms capture diminishing marginal effects at high values. This illustrates that LGO does not force threshold-based gating when the underlying clinical signal is better captured by continuous relationships.

On **eICU**, the exemplar (seed 55, rank 1, *R*^2^ = 0.808) employs six functional logistic gates covering consciousness (Glasgow Coma Scale [GCS] ≤12.3), respiration (resp. rate >18.6 min^−1^; mechanical ventilation), hemodynamics (vasopressor use; lactate >3.4 mmol/L) and a composite renal-cardiac term (HR + creatinine). For transparency, we state the full simplified equation below (all inputs are standardized *z*-scores):

(Equation 4) $$\begin{array}{cc} {\hat{y}}_{\text{eICU}}\approx & \left(g_{\text{GCS}}+g_{\text{RR}}\right)\left(g_{\text{MV}}+g_{\text{VP}}\right)-spow\left(-\frac{\sqrt{g_{\text{lac}}}}{0.02764},\;g_{\text{HR}+\text{Cr}}-0.3541\right),\\ g_{\text{GCS}} & =\sigma \left(1.313\;\left(-{GCS}_{z}+0.5767\right)\right),\;g_{\text{RR}}=\sigma \left(3.9457\;\left({RR}_{z}+1.4398\right)\right),\\ g_{\text{MV}} & =\sigma \left(3.9457\;\left({MV}_{z}+0.3814\right)\right),\;g_{\text{VP}}=\sigma \left(3.9457\;\left({VP}_{z}+0.0784\right)\right),\\ g_{\text{lac}} & =\sigma \left(2.8236\;\left({lac}_{z}+0.0784\right)\right),\;g_{\text{HR}+\text{Cr}}=\sigma \left(2.8362\;\left(0.1733\;{HR}_{z}+{Cr}_{z}+1.4398\right)\right), \end{array}$$

where *σ*(*z*) = 1/(1 + *e*^−*z*^) and spow denotes protected exponentiation (see SI, “Protected operations”). The corresponding natural-unit cut-points are GCS ≤12.3, RR > 18.6 min^−1^, MV > 0.59, VP > 0.40, and lactate >3.42 mmol/L (Table S6). The organ-system gates closely mirror the SOFA scoring dimensions, and the lactate cut-point lies between the Surviving Sepsis Campaign’s 2.0 mmol/L trigger and severe shock levels, suggesting the model targets a moderate-high acuity regime.

On **NHANES**, the exemplar (seed 34, rank 4, *R*^2^ = 0.803) is a metabolic threshold model with two active step-like gates (*a* ≈ 16, transition width ≈0.25 std) plus a constant term from a third gate that is effectively always on:

(Equation 5) $${\hat{y}}_{\text{NHANES}}\approx \sigma \left(\text{SBP}\geq 126\;\text{mmHg}\right)+\sqrt{\text{triglycerides}}+1.0+\sigma \left(\text{glucose}\geq 98\;\text{mg/dL}\right).$$

The systolic blood pressure (SBP) threshold of 126 mmHg is close to the American Heart Association Stage 1 hypertension cutoff (130 mmHg), and the fasting glucose threshold of 98 mg/dL is within 2 mg/dL of the American Diabetes Association impaired-fasting-glucose boundary (100 mg/dL). The steep gates produce near-binary switching, consistent with the LGO_hard_ design intent.

Together, these three exemplars demonstrate that LGO can discover clinically meaningful structures ranging from smooth compositions to multi-threshold gated models, adapting to the statistical properties of each dataset. For comparison, the cross-validation-optimal models (which employ 9–12 nested gates and achieve higher *R*^2^) are reported in SI Table S4, illustrating the accuracy-interpretability trade-off. The raw expressions and gate parameters are reported in SI Tables S5–S7.

#### Fairness in model complexity

One potential concern is that LGO models introduce two continuous parameters (*a*, *b*) for each gate, whereas purely algebraic SR engines only optimize constant coefficients. To examine whether these additional degrees of freedom might advantage LGO, we recomputed the model complexities by counting each gate as two extra nodes, i.e., complexity’ = node count +2 × (*#*gates). Across the ICU, eICU, and NHANES exemplars, the models contained 0, 6, and 2 active gates, respectively, so the complexity adjustment adds at most twelve nodes to an expression with 30–39 nodes. This adjustment did not appear to change the Pareto ordering of methods or the location of the knee points: LGO_hard_ achieved performance comparable to PySR and Operon at similar complexity levels. These observations suggest that the extra continuous parameters in LGO models did not substantially affect the fairness of our budget-aligned comparison.

### Overall predictive performance across datasets

**Figure 2:**
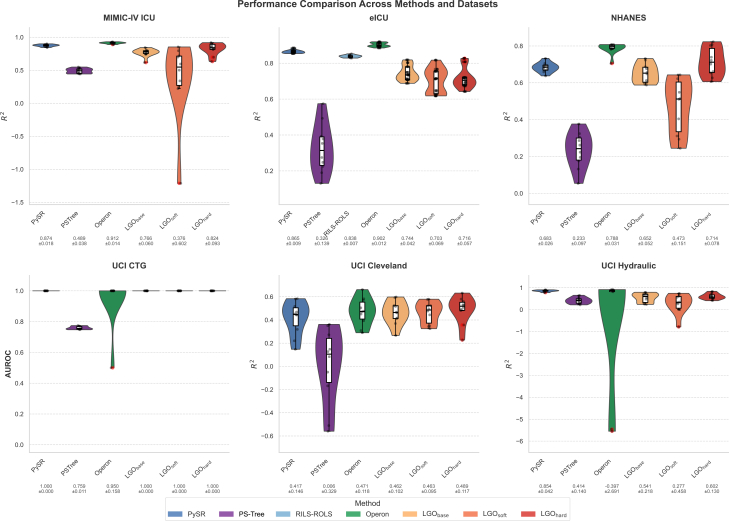
Performance comparison across methods and datasets violin plots show the distribution over up to ten random seeds per method and dataset. The black dots are individual seeds; violin width reflects probability density. For regression datasets (ICU, eICU, NHANES, Cleveland, Hydraulic), the primary metric is *R*^2^; for CTG we report AUROC. The mean and standard deviation for each violin are printed underneath (mean on the top line, ± std on the bottom line). Budgets and operator sets were aligned across SR engines where applicable (see Methods); PS-Tree uses library-default hyperparameters (Table S16).

We next situate LGO among established SR baselines. Figure 2 summarizes test performance over up to ten random seeds per method and per dataset (ICU, eICU, NHANES, CTG, Cleveland, Hydraulic), using a matched symbolic budget (Operon max evaluations 500 k; LGO/PySR populations and generations tuned to the same order of evaluations). Each violin encodes the full seed distribution, black dots mark individual runs, and the numbers underneath report mean ± standard deviation across seeds. Across datasets, high-capacity SR engines (RILS-ROLS, Operon, PySR) generally achieved the highest pure point-wise accuracy.

On ICU and NHANES, RILS-ROLS attained the highest mean *R*^2^ (0.95 ± 0.01 and 0.85 ± 0.01), followed by Operon (0.91 ± 0.01 and 0.79 ± 0.03) and PySR (0.87 ± 0.02 and 0.68 ± 0.03). On eICU, Operon led with *R*^2^ = 0.90 ± 0.01, ahead of PySR (0.86 ± 0.01) and RILS-ROLS (0.84 ± 0.01). On Hydraulic, RILS-ROLS again performed best, reaching *R*^2^ ≈ 0.95 ± 0.01. LGO variants occupy a more conservative performance envelope.

Across the three health datasets, LGO_hard_ tended to outperform LGO_soft_ and in most cases LGO_base_: on ICU, LGO_hard_ achieved *R*^2^ = 0.82 ± 0.09 versus 0.77 ± 0.06 (base) and 0.38 ± 0.60 (soft); on eICU, 0.72 ± 0.06 versus 0.74 ± 0.04 (base) and 0.70 ± 0.07 (soft); and on NHANES, 0.71 ± 0.08 versus 0.65 ± 0.05 (base) and 0.47 ± 0.15 (soft). On Cleveland, LGO_hard_ achieved the highest *R*^2^ among the methods tested (0.49 ± 0.12, slightly above Operon’s 0.47 ± 0.12), while on Hydraulic it reached moderate *R*^2^ = 0.60 ± 0.13 under the fixed budget, between LGO_base_ (0.54 ± 0.22) and PySR (0.85 ± 0.04). For the nearly linearly separable CTG classification task, LGO (all variants) and PySR saturated at AUROC/AUPRC ≈1.0, whereas Operon, PS-Tree, and RILS-ROLS showed lower performance. The spread of the violins reveals stability patterns. Operon, PySR, and RILS-ROLS showed tight dispersions on ICU/eICU/NHANES, whereas PS-Tree was more variable, especially on CTG and Hydraulic. LGO_hard_ occupied an intermediate regime: its seed-to-seed variability was smaller than that of LGO_soft_, and comparable to PySR/Operon on several tasks. These observations inform the rest of our analysis: we next focus on the primary health datasets (ICU, eICU, NHANES), where we examine predictive performance, threshold recovery in natural units, and comparison to clinical scoring baselines.

### Primary health datasets: ICU, eICU, and NHANES

#### Predictive accuracy relative to SR baselines

Tables 2, 3, and 4 report mean ± std on the held-out test split for the three health datasets. Consistent with prior work, RILS-ROLS and Operon achieved high *R*^2^ values, while PySR provided a stable baseline. On **ICU**, RILS-ROLS attained the highest accuracy (*R*^2^ = 0.95 ± 0.01), with Operon and PySR close behind. Within the LGO family, LGO_hard_ performed better than both the arithmetic-only base and LGO_soft_: it achieved *R*^2^ = 0.82 ± 0.09 with RMSE = 0.76 ± 0.19 and MAE = 0.61 ± 0.17, compared to LGO_base_ (0.77 ± 0.06) and LGO_soft_ (0.38 ± 0.60).

**Table 2:** ICU composite risk score (regression): mean ± std over 10 seeds (test split)

Method	experiment	R^2^*↑*	RMSE *↓*	MAE *↓*
PySR	base	0.87 ± 0.02	0.66 ± 0.05	0.51 ± 0.04
PS-Tree	base	0.49 ± 0.04	1.32 ± 0.03	1.06 ± 0.02
RILS-ROLS	base	**0.95** ± 0.01	**0.40** ± 0.06	**0.28** ± 0.03
Operon	base	0.91 ± 0.01	0.55 ± 0.04	0.42 ± 0.04
LGO	base	0.77 ± 0.06	0.89 ± 0.10	0.73 ± 0.09
LGO	LGO_soft_	0.38 ± 0.60	1.35 ± 0.60	1.13 ± 0.50
LGO	LGO_hard_	0.82 ± 0.09	0.76 ± 0.19	0.61 ± 0.17

On ICU, one of the ten LGOsoft seeds produced a degenerate search (R2 = −1.21); the reported mean ± std includes this run, and excluding it yields R2 = 0.55 ± 0.24.

Bold indicates the best value in each column.

**Table 3:** eICU composite risk score (regression): mean ± std over 10 seeds (test split)

Method	experiment	R^2^*↑*	RMSE *↓*	MAE *↓*
PySR	base	0.86 ± 0.01	1.13 ± 0.04	0.90 ± 0.03
PS-Tree	base	0.33 ± 0.14	2.52 ± 0.27	2.02 ± 0.21
RILS-ROLS	base	0.84 ± 0.01	1.24 ± 0.03	1.02 ± 0.02
Operon	base	**0.90** ± 0.01	**0.96** ± 0.06	**0.76** ± 0.04
LGO	base	0.74 ± 0.04	1.56 ± 0.14	1.25 ± 0.12
LGO	LGO_soft_	0.70 ± 0.07	1.67 ± 0.21	1.34 ± 0.17
LGO	LGO_hard_	0.72 ± 0.06	1.64 ± 0.19	1.31 ± 0.15

Bold indicates the best value in each column.

**Table 4:** NHANES metabolic score (regression): mean ± std over 10 seeds (test split)

Method	experiment	R^2^*↑*	RMSE *↓*	MAE *↓*
PySR	base	0.68 ± 0.03	0.75 ± 0.04	0.60 ± 0.03
PS-Tree	base	0.23 ± 0.10	1.16 ± 0.06	0.93 ± 0.03
RILS-ROLS	base	**0.85** ± 0.01	**0.51** ± 0.03	**0.40** ± 0.02
Operon	base	0.79 ± 0.03	0.61 ± 0.04	0.48 ± 0.03
LGO	base	0.65 ± 0.05	0.78 ± 0.07	0.63 ± 0.06
LGO	LGO_soft_	0.47 ± 0.15	0.96 ± 0.15	0.77 ± 0.13
LGO	LGO_hard_	0.71 ± 0.08	0.70 ± 0.09	0.54 ± 0.09

Bold indicates the best value in each column.

These results are consistent with the hypothesis that explicit (hard) gating may better capture switch-like dynamics in ICU risk prediction than purely multiplicative gates under a finite budget, though further investigation would be needed to confirm this interpretation.

To help ensure that our choice of hyperparameters for PySR and Operon does not inadvertently bias the comparison, we also re-ran the ICU experiment with the SRBench-recommended default settings for these solvers. With the defaults (31 populations of 27 individuals and 100 iterations for PySR; 100 generations for Operon) the resulting test performance was nearly identical: on the ICU composite risk task, the SRBench-default PySR achieved *R*^2^ = 0.874 ± 0.02 with RMSE = 0.657 ± 0.04 and MAE = 0.515 ± 0.04, and the SRBench-default Operon matched the aligned baseline exactly (see Table S3). These results suggest that our budget-aligned settings did not substantially affect the baselines’ predictive accuracy.

On **eICU**, which serves as an external validation cohort with a different patient mix and measurement practice, a similar pattern was observed. Under a matched 500 k evaluation budget, Operon achieved the highest mean *R*^2^(0.90 ± 0.01), followed by PySR (0.86 ± 0.01) and RILS-ROLS (0.84 ± 0.01). LGO variants were somewhat less accurate in pure prediction: LGO_hard_ attained *R*^2^ = 0.72 ± 0.06 with RMSE = 1.64 ± 0.19 and MAE = 1.31 ± 0.15, slightly above LGO_soft_ (0.70 ± 0.07) and close to LGO_base_ (0.74 ± 0.04).

On **NHANES**, RILS-ROLS again leads, with Operon and PySR following (Table 4). Within LGO, hard gating is clearly preferable to soft gating: LGO_hard_ reaches *R*^2^ = 0.71 ± 0.08 with RMSE = 0.70 ± 0.09 and MAE = 0.54 ± 0.09, outperforming both LGO_base_ (0.65 ± 0.05) and LGO_soft_ (0.47 ± 0.15). The absolute gap to the strongest baselines (RILS-ROLS 0.85 ± 0.01, Operon 0.79 ± 0.03) is moderate and, as we show next, must be weighed against the interpretability gains from explicit, unit-aware gates.

#### Generalization and train-test consistency

To assess whether these symbolic models overfit the data, we additionally evaluated all methods on their respective training splits. If a model memorizes the training data, its training error would be much lower than its test error, whereas a well-generalized model should exhibit similar performance on both splits. Across the primary health datasets (ICU, eICU and NHANES), we found that the differences between training and test performance were small: the mean absolute difference in RMSE and MAE across methods and seeds was ≲ 0.01, and the mean difference in *R*^2^ was ≲ 0.01 (see Table S24). Even the largest gap observed in the smaller Cleveland cohort (ΔRMSE ≈0.08, ΔMAE ≈0.05 and Δ*R*^2^ ≈ 0.15) remained modest relative to inter-method performance differences. These observations suggest that the unified search budgets and complexity penalties used in our experiments may help control model capacity, yielding expressions that appear to generalize reasonably to held-out data.

#### Alignment between LGO thresholds and clinical anchors

**Figure 3:**
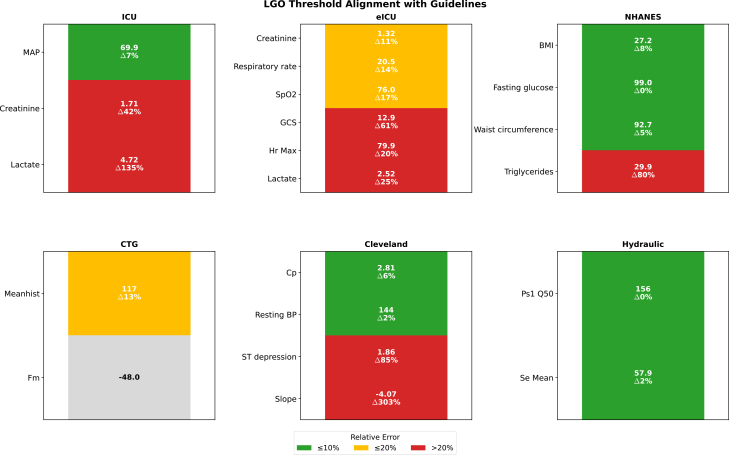
LGO threshold alignment with guidelines across datasets Each panel (ICU, eICU, NHANES, CTG, Cleveland, Hydraulic) shows anchored features for which LGO_hard_ recovers a valid median threshold in natural units. Cell color encodes the relative deviation between the LGO threshold and the curated guideline anchor (green ≤10%, yellow ≤20%, red >20%), and the text inside each cell reports the median gate (natural units) and its relative error Δ. The legend summarizes the traffic-light scheme; full numerical values for ICU/eICU/NHANES appear in Table 5, and UCI benchmark thresholds are listed in the SI.

We next examine whether LGO_hard_ identifies natural-unit thresholds that can be compared against clinical guidelines. For each dataset we retain features that have both a recovered median gate and a curated anchor, and we summarize them via a “traffic-light” deviation scale: green (≤10%), yellow (≤20%), and red (>20%) relative error. Figure 3 presents agreement heatmaps, and Table 5 reports the underlying medians and interquartile ranges (IQRs). Across ICU, eICU, and NHANES, we examined 13 anchored features. Four gates fell within ≤10% of their guideline anchors (green) and seven within ≤20% (green or yellow), leaving six “red” gates that may reflect either extreme-risk cut-points or anchors that are themselves less well-defined. These results suggest that LGO’s learned thresholds are often relatively close to domain anchors, and when they deviate, the deviations may have interpretable explanations.

**Table 5:** LGO_hard_-discovered thresholds (median [Q1, Q3]) versus domain anchors (ICU, eICU, NHANES)

Dataset	Feature	Median [Q1, Q3]	Anchor	Rel. Err.
ICU	MAP (mmHg)	69.9 [69.1, 70.0]	65.0	7.6%
ICU	creatinine (mg/dL)	1.71 [1.54, 4.86]	1.2	42.1%
ICU	lactate (mmol/L)	4.72 [2.23, 5.31]	2.0	135.9%
eICU	creatinine (mg/dL)	1.32 [0.98, 1.67]	1.5	11.7%
eICU	respiratory rate (min^−1^)	20.5	24.0	14.7%
eICU	SpO_2_ (%)	76.0 [75.7, 76.6]	92.0	17.4%
eICU	GCS (score)	12.9	8.0	61.4%
eICU	heart rate (bpm)	79.9	100.0	20.1%
eICU	lactate (mmol/L)	2.52 [1.07, 3.44]	2.0	25.8%
NHANES	BMI (kg/m^2^)	27.2	25.0	9.0%
NHANES	fasting glucose (mg/dL)	99.0 [98.0, 117.5]	100.0	1.0%
NHANES	waist circumference (cm)	92.7 [91.5, 93.4]	88.0	5.4%
NHANES	triglycerides (mg/dL)	29.9 [24.4, 29.9]	150.0	80.1%

Values are in natural units unless noted.

On **ICU**, LGO identified a MAP gate of 69.9 [69.1, 70.0] mmHg versus a 65 mmHg target (7.6% deviation; green), which could be consistent with a slightly conservative hemodynamic goal. By contrast, creatinine and lactate gates were shifted toward more severe derangements: creatinine at 1.71 [1.54, 4.86] mg/dL versus 1.2 mg/dL, and lactate at 4.72 [2.23, 5.31] mmol/L versus 2.0 mmol/L (both red). These “late” gates may function as extreme-risk alarms rather than screening thresholds, and we revisit them in the Discussion and single-feature case studies.

On **eICU**, several gates fell into the yellow band: creatinine at 1.32 [0.98, 1.67] mg/dL relative to 1.5 mg/dL (11.7%), respiratory rate at 20.5 vs. 24 min^−1^ (14.7%), and SpO_2_ at 76.0 [75.7, 76.6]% vs. a 92% anchor (17.4%). Other gates deviated more strongly: GCS at 12.9 vs. severe-impairment anchor 8.0, heart rate at 79.9 vs. 100 bpm, and lactate at 2.52 [1.07, 3.44] mmol/L vs. 2.0 mmol/L. Together, these patterns suggest that on eICU, LGO may have identified a mixture of near-anchor operating points (creatinine, respiratory rate) and earlier “warning” regimes (higher GCS, lower heart rate, mild lactate elevations) that appear to tilt toward sensitivity.

On **NHANES**, LGO_hard_ identified anthropometric and glycemic gates that appear to track cardiometabolic anchors relatively closely. Body mass index (BMI) concentrated around 27.2 versus a 25 kg/m^2^ anchor (9.0%), fasting glucose at 99.0 [98.0, 117.5] mg/dL relative to 100 mg/dL (1.0%), and waist circumference at 92.7 [91.5, 93.4] cm vs. 88 cm (5.4%). Triglycerides appeared as a clear outlier, with a median gate near 29.9 mg/dL (80.1% deviation), possibly reflecting both a highly skewed distribution and the fact that metabolic risk in this cohort may already be largely captured by waist, BMI, and glucose.

Beyond agreement, the interquartile ranges in Table 5 indicate the variability of the recovered gates across seeds. MAP, BMI, fasting glucose, and waist circumference showed narrow IQRs, suggesting relatively stable recovery; creatinine, lactate, and triglycerides had wider spreads, consistent with their red classification and with the possibility of multiple plausible operating points in the data. These red cells may not simply represent “errors” but could reflect potential extreme-risk or early-warning regimes, which we examine further via single-feature case studies in the Discussion and Supplement.

#### Comparison with clinical scoring and EBM baselines

To address requests for comparison with clinical tools, we benchmark LGO against methods used in clinical workflows:

• **AutoScore**: a semi-automatic pipeline that builds sparse point-based scores from tabular predictors.
• **Explainable Boosting Machines** (EBM, via InterpretML): a well-established generalized additive model with per-feature shape functions.

For these comparisons we binarize the composite scores into high vs. low risk (ICU: score ≥5; eICU: score ≥8; NHANES: metabolic score ≥2), and evaluate AUROC, area under the precision-recall curve (AUPRC), Brier score, F1, and accuracy over the same ten train-test splits. We use a moderate LGO budget of 30 k evaluations for all clinical-baseline experiments; the 500 k-budget SR results used for the main regression benchmarks remain in Figure 2.

On **ICU**, LGO_hard_ achieved performance broadly comparable to AutoScore under the 30 k budget, with some variation across metrics (Table 6). AutoScore attained AUROC 0.864 ± 0.010, AUPRC 0.904 ± 0.007, Brier 0.146 ± 0.005, F1 0.833 ± 0.007, and accuracy 0.784 ± 0.008. LGO_hard_ achieved AUROC 0.927 ± 0.067 and AUPRC 0.940 ± 0.057, with mean Brier score 0.119 ± 0.103 and mean accuracy 0.836 ± 0.160, though with a somewhat lower and more variable F1 (0.814 ± 0.268). Training time differed by roughly one order of magnitude: ≈0.14 s for AutoScore vs. 6.6 ± 1.8 s for LGO. These results suggest that LGO may achieve discrimination and calibration in a similar range to scoring systems while providing explicit thresholds and formulas in natural units, though at the cost of additional optimization time.

**Table 6:** MIMIC-IV ICU high-risk classification: AutoScore vs LGO_hard_ (30 k evaluation budget; mean ± std over 10 seeds)

Method	AUROC *↑*	AUPRC *↑*	Brier *↓*	F1 *↑*	Acc *↑*	Time (s) *↓*
AutoScore	0.864 ± 0.010	0.904 ± 0.007	0.146 ± 0.005	**0.833 ± 0.007**	0.784 ± 0.008	**0.14 ± 0.02**
LGO_hard_	**0.927 ± 0.067**	**0.940 ± 0.057**	**0.119 ± 0.103**	0.814 ± 0.268	**0.836 ± 0.160**	6.62 ± 1.80

Bold indicates the best value in each column.

On **ICU, eICU, and NHANES**, EBM achieved substantially higher predictive performance than LGO under the same 30 k budget (Table 7). On ICU and eICU, EBM approached ceiling-level discrimination and calibration (AUROC and AUPRC close to 1.0, Brier $\approx 10^{-2}$ or lower), likely reflecting that the composite scores are themselves derived from the same covariates. LGO_hard_ showed more modest performance: AUROC 0.843 ± 0.131 on ICU and 0.515 ± 0.294 on eICU, though with relatively high F1 and accuracy on eICU (0.945 and 0.896). On NHANES, EBM achieved AUROC 0.996 ± 0.003 and AUPRC 0.913 ± 0.054, whereas LGO_hard_ exhibited a pattern consistent with a high-specificity operating point on this imbalanced endpoint (AUROC 0.709 ± 0.221, AUPRC 0.134 ± 0.125, accuracy 0.968 ± 0.003 but low F1).

**Table 7:** High-risk classification on healthcare datasets: EBM vs. LGO_hard_ (30 k evaluation budget; mean ± std, *n* = 10)

Dataset	Method	AUROC *↑*	AUPRC *↑*	Brier *↓*	F1 *↑*	Acc *↑*
MIMIC-IV ICU	EBM	**0.998 ± 0.001**	**0.999 ± 0.001**	**0.010 ± 0.004**	**0.992 ± 0.004**	**0.990 ± 0.004**
MIMIC-IV ICU	LGO_hard_	0.843 ± 0.131	0.874 ± 0.103	0.198 ± 0.050	0.795 ± 0.065	0.685 ± 0.122
eICU	EBM	**1.000 ± 0.000**	**1.000 ± 0.000**	**0.001 ± 0.001**	**1.000 ± 0.000**	**0.999 ± 0.001**
eICU	LGO_hard_	0.515 ± 0.294	0.897 ± 0.078	0.092 ± 0.004	0.945 ± 0.000	0.896 ± 0.001
NHANES	EBM	**0.996 ± 0.003**	**0.913 ± 0.054**	**0.009 ± 0.003**	**0.788 ± 0.080**	**0.988 ± 0.004**
NHANES	LGO_hard_	0.709 ± 0.221	0.134 ± 0.125	0.030 ± 0.002	0.011 ± 0.035	0.968 ± 0.003

Bold indicates the better of the two methods for each dataset and metric.

**Figure 4:**
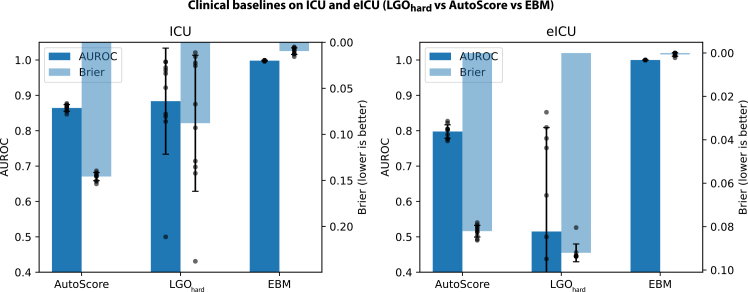
Clinical baselines on ICU and eICU (LGO_hard_ vs AutoScore vs EBM) Mean ± std performance over 10 seeds for AutoScore, LGO_hard_, and EBM on the ICU high-risk classification task, and for LGO_hard_ vs. EBM on eICU. Bars show AUROC (left axis) and Brier score (right axis, inverted); dots indicate individual seeds. LGO_hard_ reaches AutoScore-level discrimination and calibration on ICU while learning explicit thresholds; EBM provides an approximate upper bound on pure predictive performance. Error bars represent one standard deviation across n = 10 random seeds.

Figure 4 summarizes these comparisons visually. LGO_hard_ achieved AUROC/AUPRC and Brier scores broadly comparable to AutoScore on ICU, while providing explicit, unit-aware thresholds rather than point tables; EBM, as a high-capacity additive model, achieved substantially higher predictive performance but does not expose discrete cut-points in the same manner.

We further quantify these differences with paired Wilcoxon signed-rank tests across the ten train-test splits (Tables S22 and S23). On MIMIC-IV ICU, LGO_hard_ showed higher AUROC than AutoScore at the 30 k budget (0.927 ± 0.067 vs. 0.864 ± 0.010; Δ = 0.063, 95% CI [0.022, 0.098], *p* = 0.027), with 7 of 10 splits showing higher values for LGO. On eICU, a similar pattern was observed (AUROC 0.88 ± 0.08 vs. 0.80 ± 0.02; Δ = 0.082, 95% CI [0.030, 0.125], *p* = 0.020; 8/10 splits with higher LGO values). At larger budgets (100 k–500 k evaluations), AUROC differences between LGO and AutoScore became small and statistically non-significant (ΔAUROC ≈0.00–0.05, Wilcoxon *p* > 0.05), suggesting that in this regime the choice between methods may be driven primarily by interpretability considerations and workflow preferences.

For EBM vs. LGO_hard_, paired tests on the same splits indicated that EBM achieved statistically significantly higher AUROC on MIMIC-IV ICU, eICU, and NHANES (all *p* < 10^−3^, large effect sizes), consistent with EBM’s higher model capacity.

### Behavior on additional UCI benchmarks

For completeness, we also evaluated three standard UCI benchmarks.

On **CTG** (binary), LGO (all variants), and PySR reached AUROC/AUPRC ≈1.0 across seeds, suggesting near-perfect separability of the NSPbin label (the binarized fetal-state class of the CTG dataset) under the available features. Operon (AUROC ≈0.95) achieved slightly lower values, while RILS-ROLS and PS-Tree showed weaker performance (AUROC ≈0.52 and 0.76). Threshold audit on Mean hist yielded a yellow-band deviation (median LGO gate 117 vs. 135; 13.3%), which may reflect a modest shift in the operating point rather than a qualitatively different rule.

On **Cleveland** (regression), LGO_hard_ achieved the highest mean *R*^2^ among the methods tested (0.49 ± 0.12), slightly above Operon (0.47 ± 0.12) and other SR baselines. Recovered thresholds on resting blood pressure, chest-pain type (Cp) and ST depression may provide gates suitable for further audit, though some anchors (e.g., ST-segment slope) are not easily captured by a single constant and should be interpreted cautiously (Table 5; Cleveland details in supplemental information).

On **Hydraulic**, relationships appear to be predominantly smooth. RILS-ROLS achieved the highest accuracy (*R*^2^ ≈ 0.95 ± 0.01), followed by PySR (0.85 ± 0.04), while Operon showed instability under the fixed configuration. LGO_base_ and LGO_hard_ reached moderate performance (0.54 ± 0.22 and 0.60 ± 0.13, respectively), and gates focused on a small set of physical variables (e.g., Ps1 Q50, Se Mean) whose thresholds appeared to match anchor values closely (0–3% deviation). This pattern is consistent with a mechanism-selection perspective: LGO may be more useful when regime switching is plausible, while smooth primitives may suffice for globally smooth systems.

Complete mean ± std tables and per-dataset Pareto fronts (cross-validation [CV] loss vs. symbolic complexity) are reported in Tables S13–S15; Figure S1.

### Parsimony and effect of gating within the LGO family

**Figure 5:**
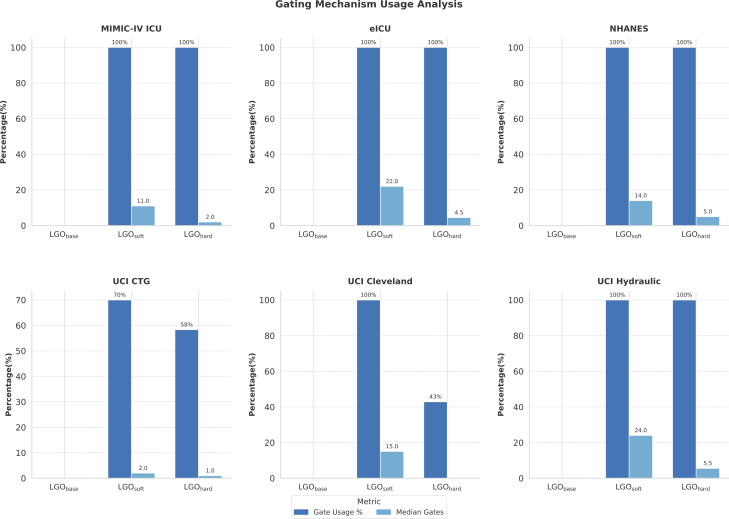
Gating mechanism usage across datasets Each panel (one per dataset): for LGO_soft_ and LGO_hard_, blue bars show gate usage % (fraction of the top-100 models that include at least one gate), and orange bars show the median number of gates per model (zeros included). Hard gates keep usage high on threshold-rich tasks (ICU, eICU, NHANES, CTG) while pruning superfluous gates on smoother problems (Cleveland, Hydraulic), yielding sparser switching structures.

A design goal of the LGO family is to express thresholded behavior with relatively few gates. We therefore quantify parsimony on the top-100 scored models per dataset (ranked by each experiment’s objective) along two axes: the fraction of models that contain at least one LGO gate (gate usage %), and the median number of gates per model (zeros included). Figure 5 and Table 8 summarize these measures.

**Table 8:** Gate usage by dataset: median number of gates among top-100 results per dataset

Dataset	LGO_soft_	LGO_hard_
ICU composite risk score	11.0	2.0
eICU composite risk score	22.0	4.5
NHANES metabolic score	14.0	5.0
UCI CTG NSPbin	2.0	1.0
UCI Heart Cleveland_num	15.0	0.0
UCI HydraulicSys fault score	24.0	5.5

Across all six datasets, LGO_soft_ used gates in nearly all models (100% gate usage; median 11–24 gates per model), whereas LGO_hard_ appeared more parsimonious. On the three health datasets, LGO_hard_ maintained 100% gate usage (ICU, eICU, NHANES) but with fewer gates: medians of 2.0 (ICU), 4.5 (eICU), and 5.0 (NHANES), compared to 11.0, 22.0, and 14.0 gates for LGO_soft_. On CTG, Cleveland, and Hydraulic, hard gating was used more selectively: a median of 1 gate on CTG, 0 on a large subset of Cleveland models, and ≈5.5 on Hydraulic. These patterns appear consistent with the threshold audit: where domain knowledge suggests sharp regimes (e.g., hypotension, hypoxemia, obesity), LGO_hard_ tended to express them via a small number of discrete gates with auditable cut-points; when the signal appeared smoother, or anchors were ambiguous, gates were often pruned or may have been repurposed as extreme-risk flags. Detailed single-feature case studies for selected gates appear in the Discussion.

To isolate the contribution of gating on accuracy, we compare three operator sets within the same engine: base (no gates), LGO_soft_, and LGO_hard_. Table 9 reports median *R*^2^ (interquartile range) over ten seeds for four regression datasets.

**Table 9:** Ablation within LGO: medians (IQR) of *R*^2^ over 10 seeds

Dataset	Base	LGO_soft_	LGO_hard_
ICU composite risk score	0.780 (0.041)	0.548 (0.443)	**0.840** (0.076)
NHANES metabolic score	0.652 (0.081)	0.512 (0.269)	**0.712** (0.131)
UCI Heart Cleveland_num	0.464 (0.110)	0.486 (0.150)	**0.518** (0.069)
UCI HydraulicSys fault score	**0.599** (0.375)	0.331 (0.535)	0.578 (0.149)

Bold indicates the highest median R^2^ for each dataset.

LGO_hard_ tended to perform better than LGO_soft_, and achieved higher *R*^2^ than LGO_base_ on three of the four tasks (ICU, NHANES, Cleveland). On ICU, the median *R*^2^ increased from 0.78 (base) to 0.84 (hard); on NHANES from 0.65 to 0.71; and on Cleveland from 0.46 to 0.52. On Hydraulic, hard gating did not appear to improve upon the smooth base (0.58 vs. 0.60 median *R*^2^) and exhibited a somewhat larger IQR, possibly reflecting occasional over-fitting when relations are globally smooth. Overall, these ablations suggest that (1) hard gates may be preferable within the LGO family on problems where thresholding appears relevant and (2) when the underlying mechanism is smooth, LGO tends to fall back to its base arithmetic behavior with only a modest reduction in accuracy.

## Discussion

### From readable formulas to auditable thresholds

SR is often positioned as a route to readable models, but readability alone may not be sufficient for scientific and regulatory practice.^3^^,^^4^^,^^20^ In high-stakes domains, stakeholders ask more pointed questions: Where is the decision boundary? In what physical units? Does it agree with domain guidelines? The LGO family proposed here attempts to address these requirements by making thresholds first-class citizens in the SR search. Instead of relying on long arithmetic compositions to approximate step-like behavior, LGO provides gating primitives with learnable location and steepness that are trained in standardized space and mapped back to natural units for audit. This approach aims to provide what we term executable interpretability: the model encodes not only a compact algebraic form, but also explicit, unit-aware cut-points that can be compared against clinical or engineering anchors.

The expanded threshold audit across six datasets (ICU, eICU, NHANES, CTG, Cleveland, Hydraulic) is consistent with this perspective (Figure 3; Table 5). Among 21 anchored feature-dataset pairs, 38% (8/21) of LGO_hard_ medians fell within ≤10% of guideline thresholds and 57% (12/21) within ≤20%, with a median relative deviation of 14%. Green cells were concentrated in ICU, NHANES, and Hydraulic, where anchors appear to closely reflect the task definition; yellow and red cells primarily arose in eICU and Cleveland, where either the label or the anchor may be less directly aligned with the prediction target. These traffic-light patterns may help turn threshold recovery into a quantitative, unit-aware audit rather than a qualitative impression.

Parsimony of gating (Figure 5; Table 8) provides a second observation. Across datasets, LGO_hard_ used fewer gates per model than LGO_soft_ (e.g., ICU 2.0 vs. 11.0; NHANES 5.0 vs. 14.0 median gates among top-100 models), while maintaining high usage rates primarily where thresholding appeared to be supported by data. This selective economy is consistent with our ablations (Table 9): hard gates performed comparably to or better than soft gates on threshold-heavy problems (ICU, eICU, Cleveland), and were near base or pruned on smoother tasks (Hydraulic).

A third perspective concerns accuracy and distributional stability (Figure 2). Although our goal is not to pursue state-of-the-art point estimates, LGO achieved performance within a reasonable range relative to established SR baselines (RILS-ROLS, Operon, PySR). The violins revealed moderate dispersion across seeds, and when some reduction in pure accuracy occurred, it may be offset by potential gains in auditability and sparsity—a trade-off that has been noted as desirable in scientific and clinical modeling.^3^

Together, these findings suggest that LGO may help reframe interpretability from a post-hoc explanation problem into a modeling constraint: searching directly in a space where threshold structure is relatively easy to express, audit, and communicate.

### Methodological significance within symbolic regression

Classical GP-based SR evolves programs over arithmetic and elementary functions.^1^^,^^2^ Recent systems (e.g., AI-Feynman, PySR) have improved efficiency and discovery quality via physics-inspired priors, equation simplification, and mixed search strategies.^28^^,^^31^^,^^37^ LGO attempts to complement this line by re-designing the primitive set: we add logistic gates—with differentiable parameters (*a*, *b*)—to make piecewise behavior more directly representable and tunable. This design is informed by decades of gating in neural networks (e.g., LSTM/GRU, mixture-of-experts),^45^^,^^46^^,^^47^ but adapted for SR where gates are symbolic nodes and their parameters can be mapped back to physical units.

A unit-aware standardize-and-invert pipeline is central to this design. All features are standardized (*z*-scores) for stable search, and learned thresholds are inverted with train-only statistics to natural units. This aims to address the common pitfall where predictions are compared across mismatched scales; we also add self-checks (RMSE ≥ MAE, internal vs. external agreement, anomaly scans) to help guard against silent failure modes. A complementary choice concerns hard versus soft gating. LGO_hard_ (threshold gate) and LGO_soft_ (multiplicative gate) realize different inductive biases: hard gates may favor sparse switching (few gates, crisp regimes), whereas soft gates may favor graded modulation. Our experiments suggest that LGO_hard_ may better match anchor-driven tasks (ICU/eICU/NHANES), whereas on globally smooth dynamics (Hydraulic), adding gates may not help—consistent with the view that the primitive set should reflect the system’s mechanism.

This perspective—curating mechanism-aware primitives—may be generalizable. Beyond thresholds, one could introduce periodic or saturation operators with parameters that map back to natural units (e.g., period in seconds, saturation level in mmol/L), potentially linking data-fit more closely to domain semantics.

### Clinical and engineering impact: From numbers to rules

Because LGO thresholds are explicitly mapped to natural units, audit becomes an arithmetic operation: compare a recovered cut-point to its guideline anchor and read off the deviation. In ICU, clinicians could verify that MAP ≈70 mmHg and lactate around the 2–5 mmol/L range are plausible escalation gates; in NHANES, systolic blood pressure near 130 mmHg, HDL around 40 mg/dL, and waist circumference in the low-90 cm range appear to align with cardiometabolic anchors, while fasting glucose around 90–100 mg/dL often surfaced an earlier turning point that may be more suitable for screening rather than diagnosis. This alignment may support clinical review, protocol integration, and post-deployment monitoring, and could help translate model outputs into unit-aware rules that stakeholders can directly discuss.^5^^,^^6^

From an engineering standpoint, gates may help formalize operating envelopes. A symbolic form like *f*(*x*) *σ*(*a*(*f*(*x*) − *b*)) exposes both the algebra and the trip point *b*, so teams could potentially wire dashboards, alarms, and acceptance tests directly to the recovered threshold in physical units. When guidance evolves (e.g., hypertension tiers), the same audit can be re-run by updating anchors; deviations then quantify how far the data-driven rule drifts from the new consensus. In this sense, LGO may help support a governance-ready loop: readable equations, unit-aware thresholds, and low-friction re-audits as standards change.

### Relation to clinical scoring systems and EBM models

Our comparison to AutoScore and EBM helps clarify where LGO may fit among clinically motivated baselines. AutoScore represents a semi-automatic implementation of the traditional points-based scoring paradigm: variables are discretized into bins, assigned integer scores, and summed to yield a risk tier. On ICU, under a matched 500 k-evaluation budget, LGO_hard_ achieved discrimination and calibration broadly comparable to AutoScore (AUROC ≈0.88 vs. 0.86; similar Brier, F1, and accuracy), while identifying thresholds that are comparable in spirit to AutoScore’s cut-points but learned directly from data. In this sense, LGO may be viewed as an approach for proposing score-like rules whose thresholds are traceable back to both the raw data distribution and domain anchors.

EBM occupies a different point in the design space. It is a high-capacity generalized additive model that achieved substantially higher performance on our binary ICU, eICU, and NHANES endpoints (AUROC ≈1.0 and very low Brier scores), and provides interpretability through per-feature shape functions and contribution plots. LGO_hard_ did not match this level of predictive performance, especially on the more imbalanced NHANES endpoint, but it offers a different form of interpretability: instead of smooth, feature-wise curves, LGO returns executable formulas with explicit gates and thresholds in physical units. In ICU and eICU, this means a clinician could both inspect a compact symbolic expression and read off concrete operating points (e.g., lactate, MAP, GCS) that might be wired into protocols, dashboards, or alarms.

Taken together, these baselines suggest the following observations. EBM achieved the highest predictive performance when accuracy is the primary objective. AutoScore provides a fast and familiar scoring-system baseline with discrete rules. LGO_hard_ occupies a different position: its accuracy was broadly comparable to AutoScore on ICU and eICU, while its potential contribution is to supply unit-aware, auditable thresholds and equations that can be directly executed and re-audited as guidelines evolve. Rather than replacing existing tools, LGO may complement them by treating threshold structure as a modeling object.

### Interpreting “yellow” and “red” cells: Screening, diagnosis, and misalignment

Traffic-light bands in Figure 3 deliberately separate three regimes: green (agreement within ≤10%), yellow (within ≤20%), and red (beyond 20%) when comparing LGO thresholds to curated anchors in natural units. Yellow-band deviations (e.g., fasting glucose and respiratory rate in NHANES and eICU) may often be best interpreted as earlier change-points rather than errors. For risk stratification and routine monitoring, a conservative turning point can be preferable to a diagnostic cutoff: the former may flag emerging risk, while the latter certifies disease. We therefore explicitly separate screening-appropriate gates—often earlier and yellow—from diagnostic thresholds—typically green—so that model interpretation may better follow clinical workflow.

Red cells warrant closer inspection. In some cases they may reflect genuine misalignment between the prediction target and the textbook anchor (e.g., composite risk scores defined as multi-factor sums rather than guideline endpoints); in others, they may signal that the anchor itself is ill-posed for a given feature (e.g., categorical encodings or non-stationary “normal ranges”). Our single-feature sanity checks are discussed in the following, may help distinguish these explanations.

### Case studies: Single-feature sanity checks

To better understand when LGO agrees or disagrees with established cut-offs, we performed single-feature sanity checks on three health datasets (ICU, eICU, NHANES). For each feature, we treat the raw measurement as a one-dimensional score, compute AUROC for the corresponding composite-risk label, and compare guideline versus LGO thresholds on the same ROC curve. Figures S2–S5 summarize these case studies, showing full distribution and ROC plots.

#### ICU lactate: An extreme-risk gate rather than a screening cutoff

In ICU, we define high risk as a composite score ≥5 (61% positives). Serum lactate was strongly associated with this outcome (AUROC = 0.83), consistent with its role as a global severity marker. The guideline cut-off at 2.0 mmol/L achieved a balanced trade-off (true positive rate [TPR] = 0.77, true negative rate [TNR] = 0.82, balanced accuracy = 0.79), whereas the LGO_hard_ median threshold at 4.7 mmol/L traded sensitivity for near-perfect specificity (TPR = 0.18, TNR = 0.98, balanced accuracy = 0.58). In other words, LGO appeared to use lactate primarily as an extreme-risk indicator: only markedly elevated lactate levels trigger the gate. This may explain the red cell for ICU lactate in Figure 3: relative to a composite risk score that already encodes multiple organ failures, the model focused on very high lactate as a signature of the most critical patients, while milder elevations may have been absorbed by other covariates.

#### eICU GCS: Shifting from coma thresholds to early neurological warning

In eICU, high risk is defined as a composite score ≥8 (59% positives). GCS showed moderate association with this label (AUC = 0.67). The classical severe-impairment threshold at GCS ≤8 operated at TPR = 0.54 and TNR = 0.73 (balanced accuracy = 0.64). LGO_hard_, by contrast, placed its median gate at GCS ≈13, treating any drop below 13 as high risk (TPR = 0.71, TNR = 0.52, balanced accuracy = 0.61). This more aggressive threshold substantially increased sensitivity at the cost of specificity, effectively treating GCS as an early-warning signal rather than a coma detector. Clinically, such a shift may be reasonable for a composite “overall deterioration” outcome, but it also illustrates how LGO can push thresholds away from traditional diagnostic cut-offs when the task rewards early detection.

#### NHANES waist circumference: Data-driven refinement around a textbook anchor

In NHANES, we mark elevated cardiometabolic burden by a metabolic score ≥2 (65% positives). Waist circumference alone was a strong predictor of this score (AUROC = 0.83). The International Diabetes Federation’s 88 cm anchor yielded TPR = 0.92 and TNR = 0.57 (balanced accuracy = 0.74). LGO_hard_ learned a slightly higher threshold at 92.7 cm, which modestly reduced sensitivity (TPR = 0.82) but improved specificity (TNR = 0.68), increasing balanced accuracy to 0.75. Here the red/green distinction is subtle: the learned gate remained in the same clinical regime as the guideline, but performed a small data-driven refinement of the screening boundary. This appears consistent with our broader finding that many green cells correspond to anchors that LGO essentially identified and then fine-tuned within a narrow range.

#### NHANES triglycerides: A failure case that flags misalignment

Triglycerides in NHANES offer a contrasting example. As a continuous score, triglycerides (TG) showed reasonable association with the metabolic-risk label (AUROC = 0.76), and the guideline cut-off at 150 mg/dL yielded a fairly balanced operating point (TPR = 0.29, TNR = 0.98, balanced accuracy = 0.64). However, LGO_hard_ placed its median gate near 30 mg/dL, effectively labeling almost everyone as high risk (TPR = 0.99, TNR = 0.05, balanced accuracy = 0.52). This striking red cell does not indicate that “very low TG is dangerous”, but rather appears to be a symptom of misalignment: in this cohort and outcome definition, triglycerides may be highly collinear with other metabolic factors, so the model benefits little from a clinically meaningful TG threshold and instead defaults to a degenerate gate. Our audit surfaces this failure explicitly, and the sanity check makes it straightforward for a reviewer to reject this particular gate while retaining others.

Taken together, these case studies suggest that LGO-recovered thresholds may fall into at least three categories: (1) anchor-confirming gates that agree with guidelines up to small refinements (e.g., NHANES waist circumference); (2) task-shifted gates that move away from diagnostic cut-offs to support screening or early warning (e.g., eICU GCS, ICU respiratory rate); and (3) misaligned or degenerate gates that should be discarded or re-anchored (e.g., NHANES triglycerides). Having explicit, unit-aware thresholds may make these distinctions more straightforward to analyze and communicate.

### Dimensional consistency and anchor curation

Auditing in natural units presupposes dimensional consistency. We standardize all continuous features in *z*-space for stable search and then invert thresholds with train-only statistics; anchors are curated with explicit units and documented sources. We avoid constant anchors for variables whose reference levels depend on age or context (e.g., counts or age-indexed normals) and record exclusions in the guideline catalog. Looking ahead, typed semantics could potentially be extended with physical-dimension types, further constraining operator compositions to unit-consistent forms and tightening the audit loop.

In addition to aggregate threshold summaries, the explicit LGO formulas in Table 1 may help make the link to clinical reasoning more tangible. On ICU and eICU, each term in the top-1 equation can be read as “one more severe state becomes true”: lactate above a shock-level value, GCS in the impaired range, SpO_2_ below a safe saturation, or creatinine above a nephrotoxicity threshold. The learned coefficients quantify how much each state moves the composite score, and the thresholds can be compared against sepsis and critical-care guidelines. On NHANES, the gates appear to align with cardiometabolic risk strata (central adiposity, hypertension, pre-diabetes), so that the symbolic model may be interpretable as a data-driven variant of familiar risk scores rather than a free-form black-box. In this sense, LGO aims to yield not just readable algebra but executable rules with explicit, unit-aware cut-points that clinicians may audit, discuss, and potentially modify.

### Guidance for practitioners

Given the retrospective scope and the limitations discussed above, we suggest using LGO as a modeling and audit aid within a broader workflow, rather than as a stand-alone decision engine.

#### When LGO may be a good fit

LGO is most appropriate when domain knowledge or operational practice suggests regime changes—for example, escalation triggers, safety limits, or qualitatively different physiological states. In such settings, LGO_hard_ can be a reasonable starting point when the goal is to surface a small number of explicit, unit-aware cut-points; LGO_soft_ can be considered when the effect is expected to vary smoothly, and discrete switching is not clinically meaningful. In either case, the presence (or absence) of gates should be treated as a property of the data and budget, not as a guarantee of underlying mechanism.

#### Anchor curation and configuration

Treat anchors as external references for reporting and audit, not as ground truth. Prefer anchors with clear unit conventions and stable clinical meaning (e.g., MAP 65 mmHg; SpO_2_ 92%; fasting glucose 100 mg/dL), and document provenance and any cohort-specific adaptations. Avoid applying single constant anchors to features whose interpretation is inherently context-dependent (e.g., categorical codes, counts, or age-indexed “normal” ranges) unless the analysis explicitly stratifies or re-encodes them. Keep the standardize-and-invert pipeline intact: compute *Z* score statistics on the training split only, optimize (*a*, *b*) in standardized space under the stated bounds, and always report recovered thresholds in natural units using train-only inversion. If gates are systematically absent under LGO_hard_, this can indicate either smooth signal or insufficient search budget; if gates proliferate (especially under LGO_soft_), consider tightening complexity penalties and verifying that the added gates improve validation loss and interpretability.

#### Audit, stress-testing, and deployment

Treat learned thresholds as hypotheses to be scrutinized. For each anchored feature, report (1) the median and interquartile range of the recovered *b*_raw_ across seeds, (2) the deviation band relative to the anchor (e.g., the traffic-light scale), and (3) simple sanity checks such as single-feature risk curves or partial-effect plots split at the recovered gate. When a learned gate deviates from an anchor, distinguish between plausible “early-warning” operating points and “late” extreme-risk cutoffs, and check whether the corresponding regime has adequate sample support. If the model is used beyond retrospective analysis, export gates as unit-aware rules with versioned feature definitions and units, and monitor *b*_raw_ under data-pipeline changes; in high-stakes settings, we suggest treating LGO as complementary to established interpretable baselines (e.g., AutoScore and EBM), so that stakeholders can compare point tables, additive shapes, and symbolic gates on the same covariates.

#### From retrospective evidence to practice

The present evidence is retrospective and limited to a small set of public cohorts. Any LGO-derived rule intended for practice should undergo domain-expert review and prospective or external validation, with attention to calibration, subgroup performance, and operational failure modes. In many workflows, a near-term role for LGO is to summarize models into a small number of explicit, unit-aware thresholds that can be discussed, challenged, and revised in guideline or quality-improvement settings, rather than to replace existing decision pathways.

### Broader outlook

LGO adds an explicit threshold primitive to symbolic regression and couples it with a standardize-and-invert reporting rule, so that recovered cut-points can be read and audited in physical units. This design provides a concrete interface between equation discovery and domain review: where the model switches, in what units, and how those values compare to an externally curated reference. In principle, the same recipe—typed parameters optimized in standardized space and then mapped back to natural units—could be extended to other structured operators whose parameters carry unit semantics (e.g., saturation or dose-response forms), but such extensions are outside the scope of the present study.

Several challenges remain. On predominantly smooth relationships, gating can be unnecessary or can over-parameterize the model; anchor sets can be incomplete, contested, or context-dependent; and our evaluation is retrospective (and single-center for ICU). We therefore view LGO as one component in a broader toolbox. Future work should emphasize uncertainty characterization for recovered gates, subgroup-aware audits, external validation, and human-in-the-loop procedures for updating anchors and feature-unit mappings. Under such practices, unit-aware symbolic gates may serve as auditable modeling artifacts that are easier to inspect, recalibrate, and govern than opaque score functions, while remaining explicit about their evidentiary limits.

### Limitations of the study

Even with unit-aware gates, LGO is not a turnkey clinical or regulatory tool. Below we summarize boundary conditions and failure modes, and how we interpret the present evidence.

#### Smooth relations and unnecessary gates

When the underlying relationship is globally smooth, introducing gates can add degrees of freedom without improving fit. In such settings, arithmetic-only SR or other strong baselines may be preferable under the same evaluation budget. In our experiments, this pattern was most apparent on smoother physical benchmarks, where the marginal value of discrete regime switching appeared limited. Practically, this supports a mechanism-aware choice of primitives: hard gates are most defensible when regime switching is plausible (alerts, safety bands, phase-like transitions), whereas smooth primitives may be more appropriate otherwise.

#### Anchor validity, feature semantics, and label design

Our threshold audit treats curated anchors as externally meaningful single cut-points. This assumption can fail: some variables do not admit a stable constant anchor (e.g., coarse categorical encodings, counts, or context-dependent “normal” ranges), and in those cases a large deviation may reflect anchor mis-specification rather than a model error. In addition, when the target is a composite score (or a proxy label derived from the same covariates), the operating point that best separates classes need not coincide with a diagnostic guideline cutoff; learned gates may shift toward earlier-warning or extreme-risk regimes. We therefore interpret anchor agreement as a sanity check for auditability and plausibility—not as a claim of clinical optimality or causal correctness.

#### Optimization budget and numerical stability

Learning gates introduces additional continuous parameters. Although strong typing, bounded parameter domains, protected operators, and local refinements are intended to improve robustness, they do not eliminate risks of local optima, brittle steepness values, or numerically inactive/degenerate gates. Budgets remain important: with too little budget, gates may be missed or poorly calibrated; with too much budget, rare-regime overfitting becomes a concern. To make these sensitivities visible, we report per-seed results, gate counts, and top-*k* pools, and we encourage readers to inspect the exported artifacts rather than relying only on aggregate summaries.

#### Aggregate summaries versus gate incidence

The main text summarizes thresholds using medians and IQRs across seeds, which does not fully convey how frequently a given gate appears (or disappears) across runs and across the top-ranked models. In practice, a threshold can be numerically stable when present yet appear only in a subset of seeds or models. We therefore log gate incidence/coverage in addition to location statistics, and recommend interpreting “a recovered threshold” together with how consistently that gate is selected.

#### Comparisons to AutoScore and EBM

Our comparisons to AutoScore and EBM are retrospective and cohort-specific, and they depend on design choices such as the evaluation budget and the binarization threshold for composite scores. Across our resampled splits, LGO_hard_ achieved AUROC in a similar range to AutoScore on ICU/eICU, with differences that vary by budget and split (see the paired tests reported in the SI). EBM, as a higher-capacity additive model, generally attained substantially higher predictive performance on these tasks. These results help position LGO as an approach for executable, unit-aware threshold reporting rather than as a replacement for stronger predictive learners.

#### Data, transportability, and deployment

Finally, all experiments are retrospective and inherit common limitations of EHR and survey analyses: confounding, dataset shift across centers, missingness and measurement-frequency artifacts, and coding practices can affect recovered thresholds. Unit-aware inversion improves interpretability, but it does not guarantee transportability across pipelines or institutions; changes in preprocessing, units, or feature definitions can shift both anchors and learned gates. Any real-world use would require prospective evaluation, domain-expert review, governance oversight, and monitoring. In this study, our goal is to make such scrutiny easier by exposing auditable, unit-aware cut-points—not to claim readiness for clinical deployment.

## Resource availability

### Lead contact

Requests for further information and resources should be directed to and will be fulfilled by the lead contact, Ou Deng (dengou@toki.waseda.jp).

### Materials availability

This study did not generate new unique reagents.

### Data and code availability

• All datasets analyzed in this study are publicly available. MIMIC-IV ICU^14^ and eICU^16^ are available from PhysioNet (data use agreement required). NHANES^18^ is available from the CDC. UCI datasets (CTG,^59^ Cleveland,^60^ Hydraulic^61^) are available from the UCI Machine Learning Repository. Accession numbers and URLs are listed in the key resources table.
• All original code is publicly available at GitHub (https://github.com/oudeng/LGO) and has been archived at Zenodo (DOI: https://doi.org/10.5281/zenodo.18712426).
• Any additional information required to reanalyze the data reported in this paper is available from the lead contact upon request.

## Acknowledgments

The work was supported in part by the 2022–2024 Masaru Ibuka Foundation Research Project on Oriental Medicine, 2020–2025 10.13039/501100001691JSPS A3 Foresight Program (grant no. JPJSA3F20200001), 2022–2024 Japan National Initiative Promotion Grant for Digital Rural City, 2023 and 2024 10.13039/501100004423Waseda University Grants for Special Research Projects (grant nos. 2023C-216 and 2024C-223), 2023–2024 10.13039/501100004423Waseda University Advanced Research Center Project for Regional Cooperation Support, and 2023–2024 10.13039/100007392Japan Association for the Advancement of Medical Equipment (10.13039/100007392JAAME) Grant.

## Author contributions

Conceptualization: O.D.; methodology: O.D.; software: O.D.; hardware: S.N. and O.D.; datasets: R.C., J.X., and O.D.; validation: O.D., R.C.; formal analysis: O.D.; investigation: O.D.; writing—original draft: O.D.; writing—review and editing: R.C., S.N., A.O., and Q.J.; visualization: O.D. and R.C.; supervision: S.N., A.O., Q.J.; funding acquisition: S.N., A.O., and Q.J.

## Declaration of interests

The authors declare no competing interests.

## STAR★Methods

### Key resources table

**Table:** 

REAGENT or RESOURCE	SOURCE	IDENTIFIER
**Deposited data**		

MIMIC-IV v3.1	PhysioNet	https://physionet.org/content/mimiciv/3.1/; https://doi.org/10.13026/kpb9-mt58
eICU Collaborative Research Database v2.0	PhysioNet	https://physionet.org/content/eicu-crd/2.0/; https://doi.org/10.13026/C2WM1R
NHANES 2017–March 2020	CDC/NCHS	https://www.cdc.gov/nchs/nhanes/
UCI CTG	UC Irvine Machine Learning Repository	https://doi.org/10.24432/C51S4N
UCI Cleveland Heart Disease	UC Irvine Machine Learning Repository	https://doi.org/10.24432/C52P4X
UCI Hydraulic Systems	UC Irvine Machine Learning Repository	https://doi.org/10.24432/C5CW21

**Software and algorithms**		

LGO (this paper)	This paper	GitHub: https://github.com/oudeng/LGO (v1.0); Zenodo: https://doi.org/10.5281/zenodo.18712426
DEAP	Fortin et al.^75^	https://github.com/DEAP/deap; https://deap.readthedocs.io/ (v1.4.3)
PySR	Cranmer.^31^	https://github.com/MilesCranmer/PySR (v1.5.9)
Operon	Burlacu et al.^29^	https://github.com/heal-research/operon
PS-Tree	Zhang et al.^30^	https://github.com/hengzhe-zhang/PS-Tree (v0.1.2)
RILS-ROLS	Kartelj and Djukanović.^32^	https://github.com/kartelj/rils-rols (v1.6.7)
AutoScore	Xie et al.^21^	https://github.com/nliulab/AutoScore
InterpretML (EBM)	Nori et al.^23^	https://github.com/interpretml/interpret (v0.7.4)
Python	SciCrunch Registry	RRID:SCR_008394; https://www.python.org/ (v3.10; baselines: 3.9.23, 3.11.13)
Anaconda	SciCrunch Registry	RRID:SCR_025572; https://www.anaconda.com/ (v25.7.0)
SciPy	SciCrunch Registry	RRID:SCR_008058; https://scipy.org/ (v1.16.1)
NumPy	SciCrunch Registry	RRID:SCR_008633; http://www.numpy.org/ (v2.3.0)
scikit-learn	SciCrunch Registry	RRID:SCR_002577; https://scikit-learn.org/ (v1.1.1; 1.7.1)
PyTorch	SciCrunch Registry	RRID:SCR_018536; https://pytorch.org/ (v2.5.1)

### Method details

#### Experimental workflow

To make the end-to-end protocol explicit, we summarize the workflow as follows:

1. **Data access and preprocessing.** Download public datasets and apply the repository’s preprocessing to obtain feature matrices/targets. Continuous features are standardized to *z*-scores using training-only statistics, and the same splits and pre-processing are reused across all methods, including clinical baselines (AutoScore and EBM).
2. **Typed SR configuration.** Instantiate a strongly typed GP (STGP) with the LGO primitive family and typed domains Feat/Pos/Thr. We study three operator sets: base (no gates), LGO_soft_ (magnitude-preserving gates), and LGO_hard_ (logistic gates that do not multiply *x*).
3. **Population search.** Run tournament selection with subtree crossover/mutation; enable a micro-mutation on each LGO node to perturb gate parameters (*a*, *b*) locally. SR engines are run under aligned evolutionary budgets.
4. **Model selection and refit.** Monitor a cross-validation (CV) proxy during search to build Pareto pools in accuracy–complexity space. Selected candidates are refit on the full training split, followed by a local coordinate descent on (*a*, *b*) with structure fixed.
5. **Threshold inversion and audit.** Map learned thresholds from *z*-space to natural units via training-only (*μ*, *σ*) and compute traffic-light deviations (green ≤10%, yellow ≤20%, red >20%).
6. **Reporting and statistics.** Report mean ± std test metrics across ten seeds and perform paired Wilcoxon signed-rank tests for LGO vs. AutoScore.

#### LGO-SR framework: typed GP with unit-aware gates

We embed LGO into an STGP pipeline implemented atop DEAP’s gp module,^75^ following typed GP principles.^26^ The type system separates feature values (Feat), positive steepness parameters (Pos), and threshold parameters (Thr). Pos is enforced via a softplus reparameterization; Thr is bounded in standardized (*Z* score) space with the aim of stabilizing search. We consider three operator configurations: base (no gates), LGO_soft_ (magnitude-preserving gates), and LGO_hard_ (pure logistic gates). Evolution runs entirely in standardized space; after training, learned thresholds are mapped back to natural units using training-only statistics. Formal operator definitions and gradients appear in supplemental information.

#### Search, model selection, and local refinement

Population-based search uses tournament selection with subtree crossover/mutation and a targeted micro-mutation that perturbs each LGO node’s (*a*, *b*) with the goal of refining gate location and steepness. A CV proxy is monitored during evolution to rank candidates and form Pareto pools (CV loss vs. symbolic complexity). Selected programs are refit on the training split, after which we apply a local coordinate descent on (*a*, *b*) with fixed structure. Unified budgets, typed operator sets, and CV-proxy settings are consolidated in Table S2.

#### Baselines and fair comparison

We compared against several SR engines with public implementations: PySR,^31^ Operon,^29^ PS-Tree,^30^ and RILS-ROLS.^32^ Budgets and operator sets were aligned across SR engines where applicable. For the clinical high-risk classification tasks, we additionally benchmarked LGO_hard_ against AutoScore^21^^,^^22^ and Explainable Boosting Machines (EBM) via InterpretML.^23^

#### Threshold recovery and audit

For each gated feature in an LGO_hard_ model, the learned threshold in standardized space is converted to natural units using training-only statistics and compared with dataset-specific anchors curated in config/guidelines.yaml. Agreement is quantified as relative deviation with traffic-light bands.

### Quantification and statistical analysis

Unless stated otherwise, we report the mean ± standard deviation across ten random seeds on a held-out test split (regression: *R*^2^, RMSE, MAE; classification: AUROC, AUPRC, Brier, F1, accuracy). Threshold summaries report medians with interquartile ranges. For the clinical baseline comparisons, we performed non-parametric paired Wilcoxon signed-rank tests with bootstrap confidence intervals and Cohen’s *d* effect sizes for LGO vs. AutoScore across multiple evaluation budgets; these results are summarized in Tables S22–S23. Statistical significance was defined as *p* < 0.05. Sample sizes (*n*) for each experiment are indicated in the figure legends and table captions.

## References

[1] Koza J. (1994). Genetic programming as a means for programming computers by natural selection. Stat. Comput..

[2] Schmidt M., Lipson H. (2009). Distilling free-form natural laws from experimental data. Science.

[3] Rudin C. (2019). Stop explaining black box machine learning models for high stakes decisions and use interpretable models instead. Nat. Mach. Intell..

[4] Molnar C. (2025). https://christophm.github.io/interpretable-ml-book.

[5] Wiens J., Saria S., Sendak M., Ghassemi M., Liu V.X., Doshi-Velez F., Jung K., Heller K., Kale D., Saeed M. (2019). Do no harm: A roadmap for responsible machine learning for health care. Nat. Med..

[6] Topol E.J. (2019). High-performance medicine: The convergence of human and artificial intelligence. Nat. Med..

[7] Singer M., Deutschman C.S., Seymour C.W., Shankar-Hari M., Annane D., Bauer M., Bellomo R., Bernard G.R., Chiche J.D., Coopersmith C.M. (2016). The third international consensus definitions for sepsis and septic shock (sepsis-3). JAMA.

[8] Rhodes A., Evans L.E., Alhazzani W., Levy M.M., Antonelli M., Ferrer R., Kumar A., Sevransky J.E., Sprung C.L., Nunnally M.E. (2017). Surviving sepsis campaign: International guidelines for management of sepsis and septic shock: 2016. Intensive Care Med..

[9] Whelton P.K., Carey R.M., Aronow W.S., Casey D.E., Collins K.J., Dennison Himmelfarb C., DePalma S.M., Gidding S., Jamerson K.A., Jones D.W. (2018). 2017 acc/aha/aapa/abc/acpm/ags/apha/ash/aspc/nma/pcna guideline for the prevention, detection, evaluation, and management of high blood pressure in adults: A report of the american college of cardiology/american heart association task force on clinical practice guidelines. Hypertension.

[10] Grundy S.M., Stone N.J., Bailey A.L., Beam C., Birtcher K.K., Blumenthal R.S., Braun L.T., de Ferranti S., Faiella-Tommasino J., Forman D.E. (2019). 2018 aha/acc/aacvpr/aapa/abc/acpm/ada/ags/apha/aspc/nla/pcna guideline on the management of blood cholesterol: A report of the american college of cardiology/american heart association task force on clinical practice guidelines. Circulation.

[11] Alberti K.G.M.M., Zimmet P., Shaw J. (2006). Metabolic syndrome—a new world-wide definition. a consensus statement from the international diabetes federation. Diabet. Med..

[12] ElSayed N.A., Aleppo G., Aroda V.R., Bannuru R.R., Brown F.M., Bruemmer D., Collins B.S., Hilliard M.E., Isaacs D., Johnson E.L. (2023). Standards of care in diabetes—2023. Diabetes Care.

[13] U.S. Food and Drug Administration, Health Canada, and Medicines and Healthcare Products Regulatory Agency (2021). https://www.fda.gov/medical-devices/software-medical-device-samd/good-machine-learning-practice-medical-device-development-guiding-principles.

[14] Johnson A.E.W., Bulgarelli L., Shen L., Gayles A., Shammout A., Horng S., Pollard T.J., Hao S., Moody B., Gow B. (2023). Mimic-iv, a freely accessible electronic health record dataset. Sci. Data.

[15] Johnson A., Bulgarelli L., Pollard T., Gow B., Moody B., Horng S., Celi L.A., Mark R. (2024). MIMIC-IV (version 3.1).

[16] Pollard T.J., Johnson A.E.W., Raffa J.D., Celi L.A., Mark R.G., Badawi O. (2018). The eicu collaborative research database, a freely available multi-center database for critical care research. Sci. Data.

[17] Goldberger A.L., Amaral L.A., Glass L., Hausdorff J.M., Ivanov P.C., Mark R.G., Mietus J.E., Moody G.B., Peng C.K., Stanley H.E. (2000). PhysioBank, PhysioToolkit, and PhysioNet: Components of a new research resource for complex physiologic signals. Circulation.

[18] Stierman B., Afful J., Carroll M.D., Chen T.-C., Davy O., Fink S., Fryar C.D., Gu Q., Hales C.M., Hughes J.P. (2021). National health and nutrition examination survey 2017–March 2020 prepandemic data files—development of files and prevalence estimates for selected health outcomes. National Health Statistics Report; no 158.

[19] DECODE Study Group the European Diabetes Epidemiology Group (2001). Glucose tolerance and cardiovascular mortality: Comparison of fasting and 2-hour diagnostic criteria. Arch. Intern. Med..

[20] Lipton Z.C. (2018). The mythos of model interpretability. Commun. ACM.

[21] Xie F., Chakraborty B., Ong M.E.H., Goldstein B.A., Liu N. (2020). AutoScore: A machine learning–based automatic clinical score generator and its application to mortality prediction using electronic health records. JMIR Med. Inform..

[22] Xie F., Ning Y., Liu M., Li S., Saffari S.E., Yuan H., Volovici V., Ting D.S.W., Goldstein B.A., Ong M.E.H. (2023). A universal AutoScore framework to develop interpretable scoring systems for predicting common types of clinical outcomes. STAR Protoc..

[23] Nori H., Jenkins S., Koch P., Caruana R. (2019). Interpretml: A Unified Framework for Machine Learning Interpretability. arXiv.

[24] Banzhaf W., Francone F.D., Keller R.E., Nordin P. (1998).

[25] Poli R., Langdon W.B., McPhee N.F. (2008).

[26] Montana D.J. (1995). Strongly typed genetic programming. Evol. Comput..

[27] La Cava W., Spector L., Danai K. (2016). Proceedings of the Genetic and Evolutionary Computation Conference.

[28] La Cava W., Burlacu B., Virgolin M., Kommenda M., Orzechowski P., de França F.O., Jin Y., Moore J.H. (2021). Contemporary symbolic regression methods and their relative performance. Adv. Neural Inf. Process. Syst..

[29] Burlacu B., Kronberger G., Kommenda M., Coello C.A.C. (2020). Proceedings of the 2020 Genetic and Evolutionary Computation Conference Companion.

[30] Zhang H., Zhou A., Qian H., Zhang H. (2022). Ps-tree: A piecewise symbolic regression tree. Swarm Evol. Comput..

[31] Cranmer M. (2023). Interpretable Machine Learning for science with pysr and symbolicregression. arXiv.

[32] Kartelj A., Djukanović M. (2023). Rils-rols: robust symbolic regression via iterated local search and ordinary least squares. J. Big Data.

[33] Sipper M., Moore J.H. (2021). Symbolic-regression boosting. Genet. Program. Evolvable Mach..

[34] Bartlett D.J., Desmond H., Ferreira P.G. (2024). Exhaustive symbolic regression. IEEE Trans. Evol. Comput..

[35] de França F.O. (2023). Alleviating overfitting in transformation-interaction-rational symbolic regression with multi-objective optimization. Genet. Program. Evolvable Mach..

[36] Błądek I., Krawiec K. (2023). Counterexample-driven genetic programming for symbolic regression with formal constraints. IEEE Trans. Evol. Comput..

[37] Udrescu S.-M., Tegmark M. (2020). Ai feynman: A physics-inspired method for symbolic regression. Sci. Adv..

[38] Cranmer M., Sanchez-Gonzalez A., Battaglia P., Larochelle H., Ranzato M., Hadsell R., Balcan M.F., Lin H. (2020). Proceedings of the 34th International Conference on Neural Information Processing Systems.

[39] Petersen B.K., Larma M.L., Mundhenk T.N. (2021). International Conference on Learning Representations. ( ICLR 2021 Oral).

[40] Kim S., Lu P.Y., Mukherjee S., Gilbert M., Jing L., Ceperic V., Soljacic M. (2021). Integration of neural network-based symbolic regression in deep learning for scientific discovery. IEEE Transact. Neural Networks Learn. Syst..

[41] Kusner M.J., Paige B., Hernández-Lobato J.M. (2017).

[42] Sahoo S., Lampert C., Martius G. (2018). http://proceedings.mlr.press/v80/sahoo18a/sahoo18a.pdf.

[43] Liu H., Simonyan K., Yang Y. (2019). International Conference on Learning Representations.

[44] Yu Z., Ding J., Li Y. (2026). Discovering network dynamics with neural symbolic regression. Nat. Comput. Sci..

[45] Hochreiter S., Schmidhuber J. (1997). Long short-term memory. Neural Comput..

[46] Cho K., van Merriënboer B., Gulcehre C. (2014). Proceedings of the 2014 Conference on Empirical Methods in Natural Language Processing (EMNLP).

[47] Jacobs R.A., Jordan M.I., Nowlan S.J., Hinton G.E. (1991). Adaptive mixtures of local experts. Neural Comput..

[48] Montúfar G., Pascanu R., Cho K., Bengio Y. (2014).

[49] Letham B., Rudin C., McCormick T.H., Madigan D. (2015). Interpretable classifiers using rules and bayesian analysis: Building a better stroke prediction model. Ann. Appl. Stat..

[50] Frosst N., Hinton G., Besold T.R., Kutz O. (2017). Proceedings of the First International Workshop on Comprehensibility and Explanation in AI and ML 2017 (CEx@AI∗IA 2017), co-located with the 16th International Conference of the Italian Association for Artificial Intelligence. CEUR Workshop Proceedings, vol. 2071.

[51] Kontschieder P., Fiterau M., Criminisi A., Bulò S.R. (2015).

[52] Ustun B., Rudin C. (2017).

[53] Brunton S.L., Proctor J.L., Kutz J.N. (2016). Discovering governing equations from data by sparse identification of nonlinear dynamical systems. Proc. Natl. Acad. Sci. USA.

[54] Raissi M., Perdikaris P., Karniadakis G.E. (2019). Physics-informed neural networks: A deep learning framework for solving forward and inverse problems involving nonlinear partial differential equations. J. Comput. Phys..

[55] Lu L., Jin P., Pang G., Zhang Z., Karniadakis G.E. (2021). Learning nonlinear operators via deeponet based on the universal approximation theorem of operators. Nat. Mach. Intell..

[56] Li Z., Kovachki N.B., Azizzadenesheli K. (2021). International Conference on Learning Representations (ICLR).

[57] Villar S., Hogg D.W., Storey-Fisher K., Yao W., Blum-Smith B., Ranzato M., Beygelzimer A., Dauphin Y., Liang P.S., Wortman Vaughan J. (2021). Proceedings of the 35th International Conference on Neural Information Processing Systems.

[58] Batzner S., Musaelian A., Sun L., Geiger M., Mailoa J.P., Kornbluth M., Molinari N., Smidt T.E., Kozinsky B. (2022). E(3)-equivariant graph neural networks for data-efficient and accurate interatomic potentials. Nat. Commun..

[59] Campos D., Bernardes J. (2000).

[60] Janosi A., Steinbrunn W., Pfisterer M., Detrano R. (1989). UCI Machine Learning Repository.

[61] Helwig N., Pignanelli E., Schtze A. (2015).

[62] Flaureau A., Weibel A., Chevallier G., Esvan J., Laurent C., Estournès C. (2022). Few-layered-graphene/zirconia composites: Single-step powder synthesis, spark plasma sintering, microstructure and properties. J. Eur. Ceram. Soc..

[63] Sobol′ I.M. (2001). Global sensitivity indices for nonlinear mathematical models and their monte carlo estimates. Math. Comput. Simulat..

[64] Herman J., Usher W. (2017). Salib: An open-source python library for sensitivity analysis. J. Open Source Softw..

[65] Glymour C., Zhang K., Spirtes P. (2019). Review of causal discovery methods based on graphical models. Front. Genet..

[66] Rubin D.B. (1976). Inference and missing data. Biometrika.

[67] Shimizu S., Inazumi T., Sogawa Y. (2011). Directlingam: A direct method for learning a linear non-gaussian structural equation model. J. Mach. Learn. Res..

[68] Grayeli A., Sehgal A., Costilla-Reyes O., Globerson A., Mackey L., Belgrave D., Fan A., Paquet U., Tomczak J., Zhang C. (2025). Proceedings of the 38th International Conference on Neural Information Processing Systems.

[69] Tian Y., Zhou W., Viscione M., Dong H., Kammer D.S., Fink O. (2025). Interactive symbolic regression with co-design mechanism through offline reinforcement learning. Nat. Commun..

[70] Raghav S.S., Kumar S.T., Balaji R., Sanjay M., Shunmuga C. (2024). Proceedings of the Genetic and Evolutionary Computation Conference Companion. GECCO ’24 Companion ( 2076–2082).

[71] Vaswani A., Shazeer N., Parmar N. (2017).

[72] Munkhdalai T., Yu H. (2017). Proceedings of the 15th Conference of the European Chapter of the Association for Computational Linguistics: Volume 1, Long Papers.

[73] Cybenko G. (1989). Approximation by superpositions of a sigmoidal function. Math. Control Signals Syst..

[74] Hornik K. (1991). Approximation capabilities of multilayer feedforward networks. Neural Netw..

[75] Fortin F.-A., De Rainville F.-M., Gardner M.-A.G., Parizeau M., Gagné C. (2012). Deap: Evolutionary algorithms made easy. J. Mach. Learn. Res..

