# Supplementary material for: Auditable unit-aware thresholds in symbolic regression via logistic-gated operators
Source: iScience. 2026 Aug 6;29(8):117092. doi: 10.1016/j.isci.2026.117092 (PMC13475696; doi:10.1016/j.isci.2026.117092)
Supplement: Supplementary file 1 [file mmc1.pdf]

iScience, Volume *29*

Supplemental information

Auditable unit-aware thresholds in symbolic regression via logistic-gated operators

Ou Deng, Ruichen Cong, Jianting Xu, Shoji Nishimura, Atsushi Ogihara, and Qun Jin

Supplemental Information

S1 Reproducibility

This section supplements the STAR Methods with additional technical details on reproducibility. For data sources, code availability, and the experimental workflow overview, see the KEY RESOURCES TABLE and METHOD DETAILS in the main text.

Determinism and seeds. As detailed in STAR Methods, each method–dataset pair is evaluated on ten random seeds (1,2,3,5,8,13,21,34,55,89). Per-seed metrics and expressions are exported in the artifact.

Budget parity. Table S1 details the hyperparameter comparison with SRBench defaults; Table S3 provides the complete operator sets and search budgets for each SR method.

SRBench defaults versus our settings. In addition to aligning budgets, we compare our PySR and Operon hyperparameters to the typical "default" settings recommended in SRBench and the official documentation for these tools. Table S1 lists a subset of key hyperparameters, the default values reported in the PySR API reference and in benchmarking literature, and the values used in our experiments. For PySR, the API reference documents defaults of `niterations`=100, `populations`=31, `population_size`=27 and `ncycles_per_iteration`=380, with the binary operator set {+,-,*,/} and no unary operators. SRBench baselines based on Operon typically employ a population of 1000 individuals, 100 generations, tournament size 5, a maximum tree length of 100 and depth 15, and a handful of local coefficient–optimization steps. Our experiments increased PySR's population size and number of populations to match the total evaluation budget (`population_size`=1000, `populations`=15, `niterations`=40) and extended the unary operator set to include {`sqrt`, `exp`, `log`, `sin`, `cos`}; for Operon we ran 500 generations with a population of 1000 individuals, keeping the tournament size at 5 but reducing the maximum expression length to 50 with the aim of encouraging sparsity. These changes were intended to ensure that PySR and Operon consume similar wall-clock budgets to the LGO variants (~5×10^5 evaluations) while remaining within the same search space defined by their native operator sets. To facilitate comparison with standard configurations, Section S2 provides results obtained by rerunning the PySR and Operon experiments with the SRBench-default settings.

Self-checks, exports, and statistical tests. The pipeline produces (i) raw and simplified expressions; (ii) per-seed test metrics for all SR methods and clinical baselines; (iii) Pareto pools with CV loss and complexity; (iv) gate-usage summaries; and (v) unit-aware thresholds in natural units (medians, IQRs, and audit summaries). The `stats_output/` directory contains the per-seed metrics and Wilcoxon signed-rank tests used in the LGO vs. AutoScore and LGO vs. EBM comparisons. Consistency checks are designed to verify that RMSE $\geq$ MAE, that internal and external metric computations agree within numerical tolerance, and that simplified expressions reproduce the raw engine outputs pointwise within a specified numeric tolerance. These exports are intended to support the summary views in the main text (performance violins, Pareto fronts, gating usage, threshold audits) and the SI tables and figures (Tables S4–S10, Figure S1).

S2 PySR and Operon baselines under SRBench default hyperparameters

To examine whether increasing PySR's population size and number of populations or Operon's number of generations affected our baselines, we re-ran the ICU composite risk task using the SRBench-recommended default hyperparameters. In particular, PySR was run with `niterations`=100, `populations`=31, `population_size`=27 and `ncycles_per_iteration`=380 while retaining the default operator set; Operon used a population of 1000 individuals for 100 generations with 5-way tournaments and a maximum tree length of 100. Table S3 compares the test performance of PySR and Operon under our budget-aligned settings with the SRBench-default configurations. The observed differences were small (<0.001 absolute change in MAE and RMSE and <0.001 in R^2), suggesting that our adjustments to match the 500k evaluation budget did not substantially affect the baseline performance in this experiment.

Table S1: Comparison of key PySR and Operon hyperparameters with SRBench recommended defaults. Related to STAR Methods and Tables 1–3.

Hyperparameter	SRBench default	This paper	Source / rationale
**PySR**			
Number of iterations	`100`	`40`	API reference default; we reduce iterations but increase populations and population size to match total evaluation budget.
Number of populations	`31`	`15`	API default uses one population per thread; our runs use 15 populations to distribute the expanded population size across islands.
Population size	`27`	`1000`	Default from API reference; we enlarge the population to explore a larger search space while maintaining equal evaluations.
Cycles per iteration	`380`	`550`	Default is 380 cycles per iteration; we set a larger value (550) following PySR guidelines to achieve $\sim 5 \times 10^5$ evaluations.
Binary operators	{+,-,*,/}	{+,-,*,/}	We keep the default binary operator set.
Unary operators	None	{`sqrt`, `exp`, `log`, `sin`, `cos`}	API default has no unary operators; we add common functions to match the operator richness of LGO and Operon.
Maximum size	`30`	`30`	We retain the default maximum tree size.
**Operon**			
Population size	1000	1000	SRBench baselines typically use 1000 individuals; we match this.
Generations	100	500	SRBench defaults run roughly 100 generations; we run 500 generations to reach our aligned evaluation budget.
Tournament size	5	5	We retain the default tournament group size of 5.
Max. expression length	$\sim$ 100	50	SRBench uses max length 100 and max depth 15; we cap length at 50 to favour sparser models without reducing evaluation budget.
Local optimization iterations	10	5	Defaults perform 10 local parameter-optimization steps; we use 5 to balance runtime and solution quality.
Allowed symbols	{`add`, `sub`, `mul`, `div`, `sqrt`, `variable`, `constant`}	same	We keep the default function set.
Max evaluations	`500000`	`500000`	Both SRBench baselines and our runs limit the number of fitness evaluations to roughly 5×10^5.

S3 Numeric coefficients and raw expressions for exemplar equations

We provide the full numeric coefficients for the exemplar equations reported in the main text. Table S4 compares the selected exemplars with the cross-validation-optimal models, and Tables S5–S7 present the raw expression strings and gate parameters for exact reproducibility.

S3.1 Exemplar model selection

For each clinical dataset, we select an *exemplar* model from the full top-k candidate pool (10 seeds × 10 candidates per seed = 100 models per dataset). The selection prioritises clinical interpretability—specifically, the presence of gated features corresponding to established clinical thresholds—while requiring competitive predictive accuracy (test R^2 within 1.5 standard deviations of the cross-seed mean).

Table S4 reports the selected exemplar alongside the cross-validation-optimal (cv-loss rank 1) model for comparison. The cv-loss-optimal models tend to employ more complex gating structures (e.g. 9 nested gates for ICU), whereas the exemplar models sacrifice some accuracy for auditability.

Protected operations. All arithmetic primitives use safe wrappers: sdiv(x, y) replaces $|y| < \epsilon$ with ϵ and maps non-finite results to 0; ssqrt$(x<0) = 0$; slog$(x\leq 0) = \log(\epsilon)$; and spow clips the exponent to $[-5, 5]$ and maps non-finite results to 0. These wrappers are essential for correct evaluation of the raw expressions; substituting standard floating-point operations changes the predicted values (and can produce $R^2 < 0$).

Table S2: Operator sets and search budgets (SR methods). Related to STAR Methods and Tables 1–3.

Method	Operator set	Budget / key options
LGO_{base}	{add, sub, mul, div, sqrt, log, pow}	$\sim 5 \times 10^5$ evaluations per seed; population 1000, 100 generations; tournament size 7; crossover 0.8; mutation 0.2; CV proxy disabled.
LGO_{soft}	LGO_{base} + soft-gating primitives	Same budget as LGO_{base}; micro-mutation probability 0.10; local optimization 60 steps.
LGO_{hard}	LGO_{base} + hard threshold primitive	Same budget as LGO_{base}.
PySR	{+, -, *, /} and unary operators {sqrt, exp, log, sin, cos}	$\sim 5 \times 10^5$ evaluations per seed; implemented via enlarged populations and iterations (see Table S1 for parameter details).
Operon	{add, sub, mul, div, sqrt} + terminals	$\sim 5 \times 10^5$ evaluations per seed; population 1000; hundreds of generations; tournament size 5; local coefficient optimization steps reduced to 5.
PS-Tree	Piecewise symbolic tree + GP refinement	Max depth 10; max leaves 32; min samples per leaf 20; population 50 (default); generations 200 (default).
RILS-ROLS	Robust SR via iterated local search	Max fitness calls 100 000; max time 3600 s; max complexity 50; sample size 1.0; complexity penalty 1×10^{-3}.

Notes: Budgets are aligned across LGO variants and comparable in scale ($\sim 5 \times 10^5$ fitness evaluations per seed) for PySR and Operon. The default vs. actual hyperparameter values for PySR and Operon are listed in Table S1, and the effect of using SRBench-default settings on performance is reported in Table S3.

Table S3: Comparison of PySR and Operon test performance under aligned versus SRBench-default hyperparameters on the ICU composite risk regression task (mean over 10 seeds). Differences (Δ) are computed as SRBench default minus aligned. Related to Table 1.

	Aligned settings (ours)			SRBench default settings		
Method	MAE	RMSE	R^2	MAE	RMSE	R^2
PySR	0.5144	0.6553	0.8743	0.5150	0.6568	0.8738
Operon	0.4163	0.5475	0.9120	0.4163	0.5475	0.9120

Table S4: Selected exemplar vs. cross-validation-optimal model for each clinical dataset.

Dataset	Model	Seed	Rank	Test R^2	Complexity	Active Gates
ICU	Exemplar	21	1	0.846	30	0
	CV-optimal	2	1	0.918	58	9 (nested)
eICU	Exemplar	55	1	0.808	39	6
	CV-optimal	2	1	0.827	87.5	10
NHANES	Exemplar	34	4	0.803	36	2
	CV-optimal	2	1	0.877	81.5	12

Tables S5–S7 present the raw symbolic expressions for each exemplar as returned by the search engine. The expressions use the internal `lgo_thre` primitive (Definition S4), which corresponds to $\sigma(a(x-b))$. All standardised thresholds b_{std} can be converted to natural units via $b_{nat} = \mu_x + b_{std}\,\sigma_x$, where (μ_x, σ_x) are the training-set mean and standard deviation of the gated feature.

S4 Logistic-Gated Operators (LGO): formal definitions and properties

We denote the logistic function by

$$\sigma(z) = \frac{1}{1+e^{-z}}.$$

Throughout, $x, y, z \in \mathbb{R}$ are feature-domain variables (Feat); $a \in \mathbb{R}_+$ is a positive steepness parameter (Pos); $b \in \mathbb{R}$ is a threshold in standardized (z-score) space (Thr). In our implementation we use $a = \text{softplus}(\tilde{a})$ with

Table S5: Raw expression and gate parameters for the ICU exemplar (Seed 21, Rank 1, $R^2 = 0.846$). The creatinine gate is numerically inactive: the surrounding $\mathrm{div}(0.041, 0) \to 1.8\times10^{14}$ renders the gate-containing term negligible, leaving a gate-free arithmetic model.

Quantity	Value
Raw expression	`add(sqrt(sub(add(sub(add(sub(sub(id(lactate_mmol_l), log(id(creatinine_mg_dl))), log(mul(0.3188, sub(0.04060, zero)))), add(sub(id(lactate_mmol_l), log(id(lactate_mmol_l))), id(idF(lactate_mmol_l)))), id(idF(idF(map_mmhg)))), id(idF(creatinine_mg_dl))), log(div(id(idF(idF(idF(map_mmhg)))), div(div(0.04060, zero), pow(mul(lgo_thre(id(creatinine_mg_dl), 7.367, 2.020), sub(0.04060, zero)), id(idF(idF(lactate_mmol_l))))))))), id(idF(idF(vasopressor_use_std))))`
Gate (inactive)	creatinine: a=7.37, b_{std}=2.02, b_{nat}≈5.07 mg/dL
Simplified form	$\hat{y} \approx \sqrt{3\,\mathrm{lac} - \ln(\mathrm{creat}) - \ln(\mathrm{lac}) - 4.35 - \mathrm{MAP}} + \mathrm{vasopressor}$

the aim of ensuring $a > 0$ and clip pre-sigmoid arguments to $[-60, 60]$ to help maintain numerical stability; b is clipped to $[-3, 3]$ in z-space.

Canonical 1-input gates (main-text Eq. (1)–(2)).

$$\mathrm{LGO}_{\mathrm{soft}}(x; a, b) = x\,\sigma\big(a(x-b)\big), \qquad \mathrm{LGO}_{\mathrm{hard}}(x; a, b) = \sigma\big(a(x-b)\big).$$

We also use the shorthand $\mathrm{gate}(u; a, b) = \sigma\big(a(u-b)\big)$ and the expression-level gate

$$\mathrm{gate\_expr}\left(f; a, b\right) = f\,\mathrm{gate}\left(f; a, b\right).$$

Pairwise and multi-input variants (as in the code).

$$\begin{aligned}
\mathrm{AND2}(x, y; a, b) &= \sigma\big(a(x-b)\big)\,\sigma\big(a(y-b)\big),\\
\mathrm{OR2}(x, y; a, b) &= 1 - \big(1 - \sigma\big(a(x-b)\big)\big)\big(1 - \sigma\big(a(y-b)\big)\big),\\
\mathrm{AND3}(x, y, z; a, b) &= \sigma\big(a(x-b)\big)\,\sigma\big(a(y-b)\big)\,\sigma\big(a(z-b)\big).
\end{aligned}$$

Limits and interpretation. Fix b. As $a \to \infty$,

$$\mathrm{LGO}_{\mathrm{hard}}(x; a, b) \to \mathbf{1}\{x > b\}, \qquad \mathrm{LGO}_{\mathrm{soft}}(x; a, b) \to x\,\mathbf{1}\{x > b\},$$

pointwise and uniformly on compact sets avoiding $x = b$. In the same limit,

$$\mathrm{AND2}(x, y; a, b) \;\to\; \mathbf{1}\{x > b,\ y > b\}, \qquad \mathrm{OR2}(x, y; a, b) \;\to\; \mathbf{1}\{x > b \text{ or } y > b\},$$

so pairwise gates approximate indicators of axis-aligned rectangles (AND) and their unions (OR). See main-text Equation (1)–(2) for the canonical 1-input gates.[1]

Gradients (used in local refinements). Let u be either a feature x or a subexpression $f(\cdot)$ and $s = \sigma(a(u-b))$. Then

$$\begin{aligned}
\frac{\partial\,\mathrm{LGO}_{\mathrm{hard}}}{\partial a} &= (u-b)\,s(1-s), & \frac{\partial\,\mathrm{LGO}_{\mathrm{hard}}}{\partial b} &= -a\,s(1-s),\\
\frac{\partial\,\mathrm{LGO}_{\mathrm{soft}}}{\partial a} &= u(u-b)\,s(1-s), & \frac{\partial\,\mathrm{LGO}_{\mathrm{soft}}}{\partial b} &= -a\,u\,s(1-s).
\end{aligned}$$

Expression-level gates use the chain rule with $u = f(\cdot)$.

Unit-aware inversion of thresholds. The standardize-and-invert pipeline is described in the main text and STAR Methods (see "Unit-aware threshold recovery" and "Threshold recovery and audit"). Here we note that for expression-level gates $\mathrm{gate\_expr}(f; a, b)$, we estimate the z-standardization of f on the training fold before applying inversion.

[1] In code, `lgo_thre` implements $\mathrm{LGO}_{\mathrm{hard}}$; `lgo` implements $\mathrm{LGO}_{\mathrm{soft}}$; `lgo_and2`, `lgo_or2`, and `lgo_and3` implement AND2, OR2, and AND3, respectively.

Table S6: Raw expression and gate parameters for the eICU exemplar (Seed 55, Rank 1, $R^2 = 0.808$).

Quantity	Value
Raw expression	`sub(mul(add(lgo_thre(sub(sub(mul(one, zero), id(gcs)), div(div(zero, one), sub(zero, -0.3808))), as_pos(pow(one, -0.00293)), -0.5767), lgo_thre(id(idF(idF(idF(resprate_max)))), 3.9457, -1.4398)), add(lgo_thre(id(idF(idF(mechanical_ventilation_std))), 3.9457, -0.3814), lgo_thre(id(idF(idF(idF(idF(vasopressor_use_std))))), 3.9457, -0.0784))), pow(div(sqrt(lgo_thre(id(idF(idF(idF(idF(lactate_mmol_l))))), 2.8236, -0.0784)), sub(-0.02764, zero)), add(lgo_thre(add(mul(lgo_thre(-2.3323, as_pos(zero), -0.0784), id(idF(idF(hr_max)))), id(idF(creatinine_mg_dl))), 2.8362, -1.4398), sub(zero, 0.3541))))`
Simplified form	$\hat{y}_{\text{eICU}} \approx (g_{\text{GCS}} + g_{\text{RR}})(g_{\text{MV}} + g_{\text{VP}}) - \operatorname{spow}\Big(-\frac{\sqrt{g_{\text{lac}}}}{0.02764},\ g_{\text{HR+Cr}} - 0.3541\Big)$, where $g_{\text{GCS}} = \sigma(1.313(-\text{GCS}_z + 0.5767))$, $g_{\text{RR}} = \sigma(3.9457(\text{RR}_z + 1.4398))$, $g_{\text{MV}} = \sigma(3.9457(\text{MV}_z + 0.3814))$, $g_{\text{VP}} = \sigma(3.9457(\text{VP}_z + 0.0784))$, $g_{\text{lac}} = \sigma(2.8236(\text{lac}_z + 0.0784))$, $g_{\text{HR+Cr}} = \sigma(2.8362(0.1733\,\text{HR}_z + \text{Cr}_z + 1.4398))$, $\sigma(z) = 1/(1 + e^{-z})$, and spow is the protected power operator (Section S3.1).

Gate	Feature	a	b_{std}	b_{nat}
1	GCS (inverted)	1.313	−0.577	GCS $\leq$ 12.3
2	resp. rate	3.946	−1.440	$> 18.6\,\text{min}^{-1}$
3	mech. vent.	3.946	−0.381	> 0.59
4	vasopressor	3.946	−0.078	> 0.40
5	lactate	2.824	−0.078	$> 3.42\,\text{mmol/L}$
6	HR + creatinine	2.836	−1.440	composite

S5 Local coordinate descent for gate parameter optimization

When the logistic gate in LGO has a large steepness a, the response $\sigma\big(a(x-b)\big)$ approaches a step function. As a result, the loss surface with respect to the threshold b tends to consist of flat plateaus separated by sharp drops; small changes far from the optimal threshold may have little effect on the loss, while small perturbations near the optimum can cause large changes. In such settings, gradient-based optimisers can become unreliable: the derivative of the sigmoid activation $\sigma(1-\sigma)$ tends to zero when the neuron saturates, so gradients may either vanish or become very large, making it difficult to find useful update steps. To address this, we employ a local coordinate descent (LCD) scheme that alternately updates the gate's slope a and threshold b while keeping all other parameters fixed. The approach involves converting each 1-D subproblem into a direct search over a finite set of candidate values.

S5.1 Algorithm

Let $\mathcal{T}$ be a fixed symbolic tree with m logistic gates. For gate j with parameters (a_j, b_j), we denote its input feature by x_j. The LCD procedure refines the top $k_{\text{lcd}} = 6$ candidates from the genetic search as follows:

1. **Fix the symbolic structure.** Only the continuous gate parameters (a_j, b_j) are updated; the tree topology remains frozen.
2. **Alternate between b-updates (even steps) and a-updates (odd steps).**
   - *Even steps:* For each threshold b_j, sample a candidate $b'_j = b_j + \varepsilon$ where $\varepsilon \sim \mathcal{N}(0, 0.25^2)$ in standardised units. Evaluate 5-fold cross-validation loss with (a_j, b'_j) and accept the update only if the loss decreases.
   - *Odd steps:* For each steepness a_j, compute the cross-validation loss for each candidate $a'_j = f \times a_j$ with $f \in \{0.8, 1.2, 2.0, 3.0\}$, and accept the best-improving factor.
3. **Repeat** for up to S steps. If no improvement is found for 15 consecutive steps, stop early.

Table S7: Raw expression and gate parameters for the NHANES exemplar (Seed 34, Rank 4, $R^2 = 0.803$).

Quantity	Value
Raw expression	`add(lgo_thre(id(idF(idF(idF(idF(idF(idF(systolic_bp))))))),`<br>`16.238, as_thr(sqrt(zero))), div(add(sqrt(id(triglycerides)),`<br>`add(lgo_thre(pow(id(waist_circumference), div(div(one,`<br>`add(id(gender_std), sqrt(zero))), sub(sub(mul(one, one),`<br>`id(gender_std)), id(waist_circumference)))), 16.238, -0.4810),`<br>`lgo_thre(pow(id(idF(idF(idF(fasting_glucose)))), div(pow(one, mul(one,`<br>`one)), one)), 16.238, -0.4275))), pow(div(one, one), mul(one, one))))`

Gate	Feature	a	b_{std}	b_{nat}
1	systolic BP	16.24	0.000	≥ 126.3 mmHg
2	waist (gender)	16.24	-0.481	always ON*
3	fasting glucose	16.24	-0.428	≥ 98.1 mg/dL

*Gate 2 input: pow(waist, 1/[gender × (1 − gender − waist)]), which produces NaN for most values → `nan_to_num` → 0.0 → $\sigma(16.24 \times 0.48) \approx 1.0$.

Table S8 reports the key LCD hyperparameters.

Table S8: LCD hyperparameters used in all experiments.

Parameter	Value	Description
k_{lcd}	6	Number of top-k models refined by LCD
S	150	Maximum LCD iterations
σ_{jitter}	0.25	Gaussian noise std for b update (standardised units)
Scaling factors	{0.8, 1.2, 2.0, 3.0}	Candidate multiplicative factors for a update
Patience	15	Early stopping: halt after 15 steps without improvement
CV folds	5	Cross-validation folds for candidate evaluation

This procedure requires only forward evaluations of the loss function and does not depend on gradient information; therefore it may remain more stable than gradient-based methods when the loss landscape is piecewise flat. Because the LCD steps are applied only after the genetic search has produced a promising tree structure, they consume a relatively small fraction of the overall budget and do not alter the complexity metric used to rank solutions.

S5.2 Discussion

The LCD approach can be interpreted as a form of alternating minimization on a non-smooth objective. In contrast to algorithms such as BFGS or Adam that attempt to adjust all continuous parameters simultaneously, LCD exploits the fact that, conditional on a fixed structure, each gate parameter can be updated by a one-dimensional stochastic search. This is reminiscent of the coordinate-wise minimization strategies used for ℓ_1-penalized regression and other convex problems. By decoupling the optimisation of (a_j, b_j) and using Gaussian perturbations for thresholds, LCD may help traverse the plateau-like regions caused by saturating logistic gates and may reduce the impact of vanishing gradients that can hamper standard gradient descent methods. The multiplicative scaling of the steepness parameter is motivated by the observation that a controls the sharpness of the transition and is naturally updated on a multiplicative (log) scale rather than an additive one.

S6 Top-1 and Top-k expressions

S6.1 From raw engine strings to the Simplified Expression

All engines output raw, typed strings with ephemeral constants (e.g., `add`, `mul`, `lgo_hard`, `gate_expr`). To render the human-readable equations reported in the main text, we apply a deterministic and auditable pipeline:

(i) **Parsing and typing.** Raw strings are parsed into typed ASTs with three disjoint domains: `Feat` (feature values), `Pos` (positive steepness), and `Thr` (thresholds).

(ii) **Constant folding and sanitization.** Algebraic constants are folded; numerically tiny subtrees are collapsed; denormals are clipped. Printing precision is *unit-aware* for display (e.g., mmHg: 1 decimal; mmol/L: 1 decimal; mg/dL: integer), while evaluation retains full precision.

(iii) **Canonical rewrites (sound under typing).** We apply a small rewrite set (Table S9): neutral/absorbing removals ($\texttt{add}(x,0)\to x$, etc.), associativity flattening, commutative sorting, and domain-safe identities ($\log(\exp x)\to x$, etc.).

(iv) **Gate compaction and unit-aware thresholds.** $\texttt{lgo\_hard}(x;a,b)\mapsto \mathrm{gate}(x)$, $\texttt{lgo\_soft}(x;a,b)\mapsto \mathrm{gate}(x)\cdot x$, $\texttt{gate\_expr}(f;a,b)\mapsto \mathrm{gate}(f)\cdot f$. The learned threshold b_z is mapped to natural units by $\hat{b}_{\mathrm{raw}} = \mu_x + \sigma_x\, b_z$ using *training-only* statistics (μ_x, σ_x).

(v) **Readability passes.** Factor repeated gates, linearize sums/products, and merge near-duplicate gates of the same feature when thresholds differ by less than a small tolerance (we keep the median).

(vi) **Numeric equivalence check.** We require identical external metrics on the test split and a strict pointwise tolerance $\max_i |\hat{y}_i^{\mathrm{raw}} - \hat{y}_i^{\mathrm{simp}}| < 10^{-9}$. Expressions that fail this check are left less simplified and flagged in the SI artifact.

In the health datasets, this pipeline aims to convert a raw program into a more compact representation: a small number of gates in z-space are mapped to cut-points in familiar clinical units (mmHg, mmol/L, kg/m^2, ...), each multiplied by a learned weight. By contrast, arithmetic-only engines (PySR, Operon) produce smooth combinations of the same covariates. Their effects are interpretable as shapes (e.g. monotone increases), but they do not directly expose named thresholds in the same manner.

Table S9: Canonical rewrite rules used in expression simplification. Related to STAR Methods.

Rule	Condition / Note
$\texttt{add}(x,0)\to x,\ \texttt{mul}(x,1)\to x,\ \texttt{mul}(x,0)\to 0$	Neutral/absorbing removal
$\texttt{div}(x,1)\to x,\ \texttt{pow}(x,1)\to x,\ \texttt{pow}(x,0)\to 1$	Domain valid
$\texttt{sqrt}(\texttt{pow}(x,2))\to x$	Sound when $x\geq 0$ (`Pos`/non-neg.)
$\log(\exp x)\to x,\ \exp(\log x)\to x$	Domain preserved ($x>0$)
Flatten `add`/`mul`; sort commutative args	Canonical, improves readability
$\texttt{lgo\_soft}(x;a,b)\to \mathrm{gate}(x)\cdot x$	Gate compaction
$\texttt{lgo\_hard}(x;a,b)\to \mathrm{gate}(x)$	Probabilistic gate
$\texttt{gate\_expr}(f;a,b)\to \mathrm{gate}(f)\cdot f$	Expression-level gating

S6.2 Worked examples: simplification of the NHANES exemplar

We illustrate the constant-folding and simplification process using the NHANES exemplar (Seed 34, Rank 4), which has a clear gate structure and a tractable expression length.

Step 1: Constant folding. We apply symbolic identities: `zero`→0, `one`→1, `id`(x)→x, `idF`(x)→x.

- `as_thr(sqrt(zero))` → `clip(0, −3, 3)` → 0.
- `pow(div(one,one), mul(one,one))` → $\mathrm{pow}(1,1)=1$.
- $\texttt{div}(\cdots,1)$ → the numerator itself.

Step 2: Gate compaction.

- Gate 1: `lgo_thre(systolic_bp, 16.238, 0)` $\rightarrow \sigma(16.24 \times (\text{SBP}_{\text{std}} - 0))$. b_{std}=0 means the threshold equals the training mean: $b_{\text{nat}} = \mu_{\text{SBP}} = 126.3\,\text{mmHg}$. With a=16.24, the transition width is $\approx 4/a \approx 0.25$ std, producing near-step switching.
- Gate 2: The input $\text{pow}(\text{waist}, 1/[\text{gender} \times (1 - \text{gender} - \text{waist})])$ produces NaN for most patients because the exponent denominator crosses zero. The protected evaluator maps NaN→0, yielding $\sigma(16.24 \times (0 + 0.481)) = \sigma(7.81) \approx 1.0$. This gate is *always on* and absorbs into a constant +1.
- Gate 3: `lgo_thre(fasting_glucose, 16.238, −0.4275)` $\rightarrow$ fires when glucose exceeds $b_{\text{nat}} = \mu + (-0.428)\,\sigma = 98.1\,\text{mg/dL}$.

Step 3: Final simplified form.

$$\hat{y}_{\text{NHANES}} \approx \underbrace{\sigma(\text{SBP} \geq 126\,\text{mmHg})}_{\text{Gate 1}} + \sqrt{\text{triglycerides}} + \underbrace{1.0}_{\text{Gate 2}} + \underbrace{\sigma(\text{glucose} \geq 98\,\text{mg/dL})}_{\text{Gate 3}}.$$

The SBP threshold (126 mmHg) is close to the AHA Stage 1 hypertension cutoff (130 mmHg), and the glucose threshold (98 mg/dL) falls within 2 mg/dL of the ADA impaired-fasting-glucose boundary (100 mg/dL). The triglyceride contribution enters via a square-root transform, providing a smooth, diminishing-returns relationship without an explicit threshold.

ICU exemplar (gate-free). On the ICU cohort, the exemplar (Seed 21, Rank 1) contains a creatinine gate in the raw expression, but the surrounding `div(0.04060, zero)` produces $\sim 1.8 \times 10^{14}$, which makes the gate-containing term negligible after constant folding. The simplified model is a gate-free arithmetic composition: $\hat{y} \approx \sqrt{3\,\text{lac} - \ln(\text{creat}) - \ln(\text{lac}) - 4.35 - \text{MAP}} + \text{vasopressor}$. This illustrates that LGO does not force gating when continuous relationships suffice.

eICU exemplar (6 gates). The eICU exemplar (Seed 55, Rank 1) retains six functional gates covering four organ systems (see Table S6). The multiplicative structure—$[\text{consciousness} + \text{respiration}] \times [\text{support}] \times f(\text{metabolism}, \text{renal-cardiac})$—produces interactions between organ-system gates, consistent with the clinical observation that multi-organ involvement amplifies risk non-additively.

S6.3 Lightweight top-k audit

To complement the top-1 exemplars, we summarize the top-k candidate pool per dataset (default $k = 100$) using the aggregated CSV files shipped with the artifact. Here we focus on the three clinically oriented cohorts (ICU, eICU, NHANES), where gates may have more direct clinical interpretation.

- **Gate selectivity and sparsity.** We report (i) the fraction of models that contain at least one gate (% with gates) and (ii) the median number of gates per model (zeros included). Table S10 consolidates these statistics for ICU, eICU and NHANES. The pattern is consistent with the main text: LGO_{hard} maintained high gate usage on these datasets but with fewer gates per model than LGO_{soft}, which tended to gate a larger number of features. This suggests that hard gates may concentrate on a smaller set of variables.
- **Feature-wise gate incidence and threshold agreement.** We list, for each cohort, the most frequently gated features (Top-3 to Top-5), the median threshold in natural units (with IQR), and the share of models whose gates fall within $\leq 10\%$ / $\leq 20\%$ of guideline anchors. Table S11 summarizes ICU and eICU side-by-side; Table S12 summarizes NHANES. No additional plotting is required; these tables provide a lightweight, reproducible summary of which features were most frequently gated.

Across the three health datasets, LGO_{hard} exhibited relatively selective gate usage and a sparser switching structure than LGO_{soft}. On ICU and eICU, the most common gates appeared on variables that may correspond to clinically relevant states: elevated lactate, impaired renal function (creatinine), low MAP, the

use of vasopressors and mechanical ventilation, depressed GCS, tachypnea and low SpO_2. On NHANES, gates clustered on anthropometric and metabolic variables associated with cardiometabolic risk (waist circumference/BMI, systolic blood pressure, fasting glucose, triglycerides), potentially reflecting obesity, hypertension and hyperglycaemia regimes. The thresholds are mapped back to natural units and compared against guideline values, so each gate can be described as "this model treats lactate $\geq$ shock-range" or "waist circumference in the central-obesity range", rather than as a change in a continuous score. These pool-level patterns appear consistent with the top-1 expressions and the main-text threshold audit.

Table S10: Top-k gate usage and sparsity on the three primary health datasets (ICU, eICU, NHANES; $k = 100$). For each experiment, we report the fraction of top-k models that contain at least one gate (% with gates), the median number of gates per model (zeros included), and the median cross-validation loss and symbolic complexity. LGO_{hard} keeps gate usage high where thresholding is useful while using substantially fewer gates than LGO_{soft}. Related to Table 7 and Figure 5.

Dataset	Experiment	Top-k	Gate usage (%)	Median # gates	Complexity (median)	CV loss (median)
ICU	base	100	0.0	0.0	36.00	0.6447
ICU	lgo_hard	100	100.0	2.0	45.50	0.5326
ICU	lgo_soft	100	100.0	11.0	82.30	1.0376
eICU	base	100	0.0	0.0	50.00	2.3961
eICU	lgo_hard	100	100.0	4.5	60.00	2.5654
eICU	lgo_soft	100	100.0	22.0	130.35	2.4859
NHANES	base	100	0.0	0.0	36.00	0.5941
NHANES	lgo_hard	100	100.0	5.0	53.00	0.4583
NHANES	lgo_soft	100	100.0	12.5	72.15	0.7449

Table S11: ICU and eICU (Top-k) most frequently gated features with unit-aware thresholds (natural units). For each feature we report how often it is gated in the top-k candidate pool for LGO_{hard} (Gate cnt, number of models N, and percentage), together with the median gate threshold and interquartile range in natural units, and the gate type. On ICU, gates appeared most frequently on variables such as lactate, creatinine, MAP, and vasopressor use; on eICU they additionally included ventilation status and SpO_2. These patterns may be consistent with clinical variables commonly associated with high-risk states. Related to Table 4 and Figure 3.

Dataset	Feature	Unit	Gate	Models w/ gate		Threshold			Gate
			cnt	N	%	Median	Q1	Q3	type
ICU	creatinine_mg_dl	mg/dL	44	100	44	1.71	1.54	4.86	lgo_thre
ICU	lactate_mmol_l	mmol/L	52	100	52	4.72	2.23	5.31	lgo_thre
ICU	map_mmhg	mmHg	11	100	11	69.95	69.08	69.98	lgo_thre
ICU	vasopressor_use_std	(std)	22	100	22	0.59	0.59	0.59	lgo_thre
eICU	lactate_mmol_l	mmol/L	77	100	77	2.52	1.07	3.44	lgo_thre
eICU	mechanical_ventilation_std	(std)	57	100	57	0.52	0.48	0.60	lgo_thre
eICU	vasopressor_use_std	(std)	52	100	52	0.40	-0.18	0.40	lgo_thre
eICU	creatinine_mg_dl	mg/dL	25	100	25	1.32	0.98	1.67	lgo_thre
eICU	spo2_min	%	20	100	20	76.00	75.67	76.59	lgo_thre

The learned gating structures showed some differences between datasets: MIMIC-IV ICU models more frequently gated on lactate with thresholds in the elevated range and on MAP near conservative hemodynamic targets, whereas eICU models exhibited lower lactate thresholds, SpO_2 cutoffs in the severe hypoxemia range, and frequent gating on mechanical ventilation and vasopressor status—patterns that may reflect differences in patient populations or clinical practices between institutions.

Table S12: NHANES (Top-k) most frequently gated features with unit-aware thresholds (natural units). Gates fall on classic cardiometabolic risk factors: central obesity (waist circumference), elevated systolic blood pressure, impaired fasting glucose, low HDL, and related demographic covariates. Thresholds are expressed directly in cm, mmHg, mg/dL, etc., so each gate can be read as an explicit rule such as "waist circumference in the abdominal-obesity range" or "fasting glucose in the pre-diabetes range". Related to Table 4 and Figure 3.

Dataset	Feature	Unit	Gate	Models w/ gate		Threshold			Gate
			cnt	N	%	Median	Q1	Q3	type
NHANES	waist_circumference	cm	31	31	100	93.94	91.67	98.02	lgo_thre
NHANES	systolic_bp	mmHg	28	40	100	128.34	128.34	128.89	lgo_thre
NHANES	fasting_glucose	mg/dL	24	37	100	85.45	74.54	95.40	lgo_thre
NHANES	gender_std	(std)	21	20	100	0.27	0.27	0.27	lgo_thre
NHANES	hdl_cholesterol	mg/dL	3	3	100	39.65	39.65	39.65	lgo_thre

S7 Anchors and unit metadata

Purpose. To facilitate unit-aware comparison of learned thresholds against domain references, we curate anchors (reference cut-points) and units in a single YAML file. `config/guidelines.yaml` contains entries for the three main health datasets (ICU, eICU, NHANES) as well as the UCI benchmarks. These anchors provide reference values such as MAP 65 mmHg, lactate 2.0 mmol/L, GCS 8, SpO_2 92%, or fasting glucose 100/126 mg/dL. Sources include Surviving Sepsis Campaign guidance for ICU/eICU anchors and ACC/AHA / ADA / IDF criteria for cardiometabolic anchors in NHANES. Exact references are listed in the main text.

Schema. Entries are grouped by dataset name, with an optional `global` section for anchors reused across datasets. Each feature entry binds a processed feature name to a unit string, anchor value, and an optional note:

```yaml
datasets:
  ICU_composite_risk_score:
    map_mmhg:
      unit: "mmHg"
      anchor: 65.0
      note: "MAP target (Surviving Sepsis Campaign)"
    lactate_mmol_l:
      unit: "mmol/L"
      anchor: 2.0
      note: "Hyperlactatemia threshold"
  eICU_composite_risk_score:
    gcs_min:
      unit: "score"
      anchor: 8.0
      note: "Severe neurologic impairment (GCS $\le$ 8)"
    spo2_min:
      unit: "%"
      anchor: 92.0
      note: "Hypoxemia alarm threshold"
  NHANES_metabolic_score:
    systolic_bp:
      unit: "mmHg"
      anchor: 130.0
      note: "ACC/AHA elevated BP"
    fasting_glucose:
      unit: "mg/dL"
      anchor: 100.0
      note: "ADA impaired fasting glucose"
```


Unit strings follow the conventions used in the main text (e.g., mmHg, mmol/L, mg/dL, cm). Standardized indicators (e.g., `gender_std`) are documented with `"(std)"` as unit and a short note explaining the coding.

Curation and provenance. For ICU and eICU, anchors are drawn from critical-care practice (hemodynamic targets, neurologic cut-points, hypoxemia alarms, renal dysfunction thresholds). Examples include MAP 65 mmHg, GCS 8, SpO_2 92%, respiratory rate 24 min^{-1}, creatinine 1.2–1.5 mg/dL, lactate 2.0 mmol/L. For NHANES, anchors follow ACC/AHA blood-pressure guidance, ADA glucose criteria and IDF/ATP-III definitions of central obesity and dyslipidemia (e.g., waist circumference, HDL, triglycerides). UCI benchmarks use anchors only where a stable, dataset-independent reference exists (e.g., blood-pressure levels, obvious physical ranges); we *avoid* constant anchors for categorical codes, counts, or clearly age-indexed normals (e.g., some Cleveland features), and note those omissions in the YAML.

Integration in audit. The threshold inversion and traffic-light deviation calculation are detailed in STAR Methods ("Threshold recovery and audit"). Here we document the anchor curation decisions and coverage validation.

Coverage and maintenance. We validate that every anchor in `guidelines.yaml` refers to an existing feature in the processed dataset and emit a coverage report listing (i) anchored features and (ii) present but unanchored features. Anchors are versioned within the repository; updates require a short provenance note and citation in the YAML. This approach is intended to make it clearer when a guideline change (e.g., updated hypertension stages) may be responsible for a shift in the audit, rather than a change in the learned model.

Limitations. Anchors encode *reference* cut-points, which may differ from *operational* alerting thresholds or locally preferred practice. For example, ICU respiratory-rate alarms may be set around 24 min^{-1}, while clinical concern can start earlier; GCS 8 marks severe impairment, but clinicians often respond to more subtle deteriorations. We therefore present both the anchor and the learned threshold distribution (median/IQR), and we suggest interpreting yellow/red cells either as (i) potentially conservative or extreme-risk gates (e.g., elevated lactate, low SpO_2, high BMI/waist), or as a signal that the underlying anchor may itself

be context-dependent (e.g., age-indexed normals in Cleveland). These considerations are discussed in the main-text threshold audit and case-study sections.

S8 Pareto and complexity

Definitions. *Complexity* counts typed primitive nodes (operators, variables, constants) in the final expression. *CV loss* denotes the shared cross-validation proxy used during search (folds, warm-up, and subsampling exactly as in the main configuration). For each method and dataset we form Pareto fronts in the two-dimensional space (CV loss, complexity).

Protocol. For every dataset (ICU, eICU, NHANES, CTG, Cleveland, Hydraulic) and every method, we collect the top-k candidates ($k = 100$) by the engine's internal score, recompute CV loss with the shared proxy, and then discard dominated points. The surviving points form a combined Pareto front across methods. In Figure S1, marker color encodes the method and the axes show complexity and CV loss; all methods are evaluated under the same CV proxy and complexity definition.

Reading guide. Points toward the lower-left corner (low CV loss at low complexity) represent accuracy–simplicity trade-offs that may be of interest. On the three health datasets (ICU, eICU, NHANES), LGO_{hard} and LGO_{base} populated the low-loss region at intermediate complexities: they fell between simpler but higher-loss PS-Tree models and more complex arithmetic baselines, while LGO_{soft} tended to produce longer expressions for comparable loss. On NHANES, hard-gated models achieved near-front CV losses at lower complexity than some high-loss, high-complexity Operon candidates. On CTG, all methods reached near-zero CV loss and the fronts were essentially saturated, consistent with the near-separable nature of the task. On Cleveland, LGO_{hard} extended the front to lower CV losses at moderate–high complexity, while PySR points offered slightly higher loss at lower complexity. On Hydraulic, relationships appeared predominantly smooth: PySR and RILS-ROLS traced the front at low complexity, while LGO variants either pruned gates and behaved similarly to their arithmetic base or occupied higher-complexity points with somewhat higher loss.

Overall, these fronts are consistent with the observations in the main text: LGO_{hard} did not occupy the extreme low-loss / very-high-complexity corner, but instead produced models in a region that may trade some accuracy for sparser, gate-based structure and thresholds expressed in natural units.

S9 UCI benchmarks

Task characterization. The three UCI benchmarks represent different task characteristics. CTG (NSPbin) is a binary classification task based on histogram features that appears to be nearly linearly separable. Cleveland (num) is a regression problem with mixed clinical covariates and a relatively small sample size. HydraulicSys fault score is a regression task where sensor–response relationships appear predominantly smooth.

Summary of results. On **CTG**, multiple engines (PySR, Operon, all LGO variants) reached AUROC and AUPRC at ≈ 1.0 with similar Brier scores (Table S13), consistent with near-perfect separability under simple formulas. In this regime, LGO behaved similarly to an arithmetic SR method: it produced compact expressions and used few gates, which may reflect that straightforward monotone scores suffice for this task.

On **Cleveland**, LGO_{hard} achieved mean R^2 comparable to other SR baselines (0.48 ± 0.12), similar to Operon (0.48 ± 0.11) and somewhat higher than PySR (0.42 ± 0.11) under the shared budget (Table S14). The corresponding expressions placed gates on variables such as resting blood pressure, ST-segment depression, and exercise-induced angina, providing thresholds that could potentially be compared against clinical references, while purely arithmetic baselines encoded similar information through smoother nonlinearities.

On **Hydraulic**, the underlying relationships appeared predominantly smooth. As might be expected, RILS-ROLS and PySR achieved high R^2 (0.95 and 0.74 on average) and low complexity (Table S15). LGO_{base} and LGO_{hard} reached moderate performance but generally showed lower accuracy than smooth

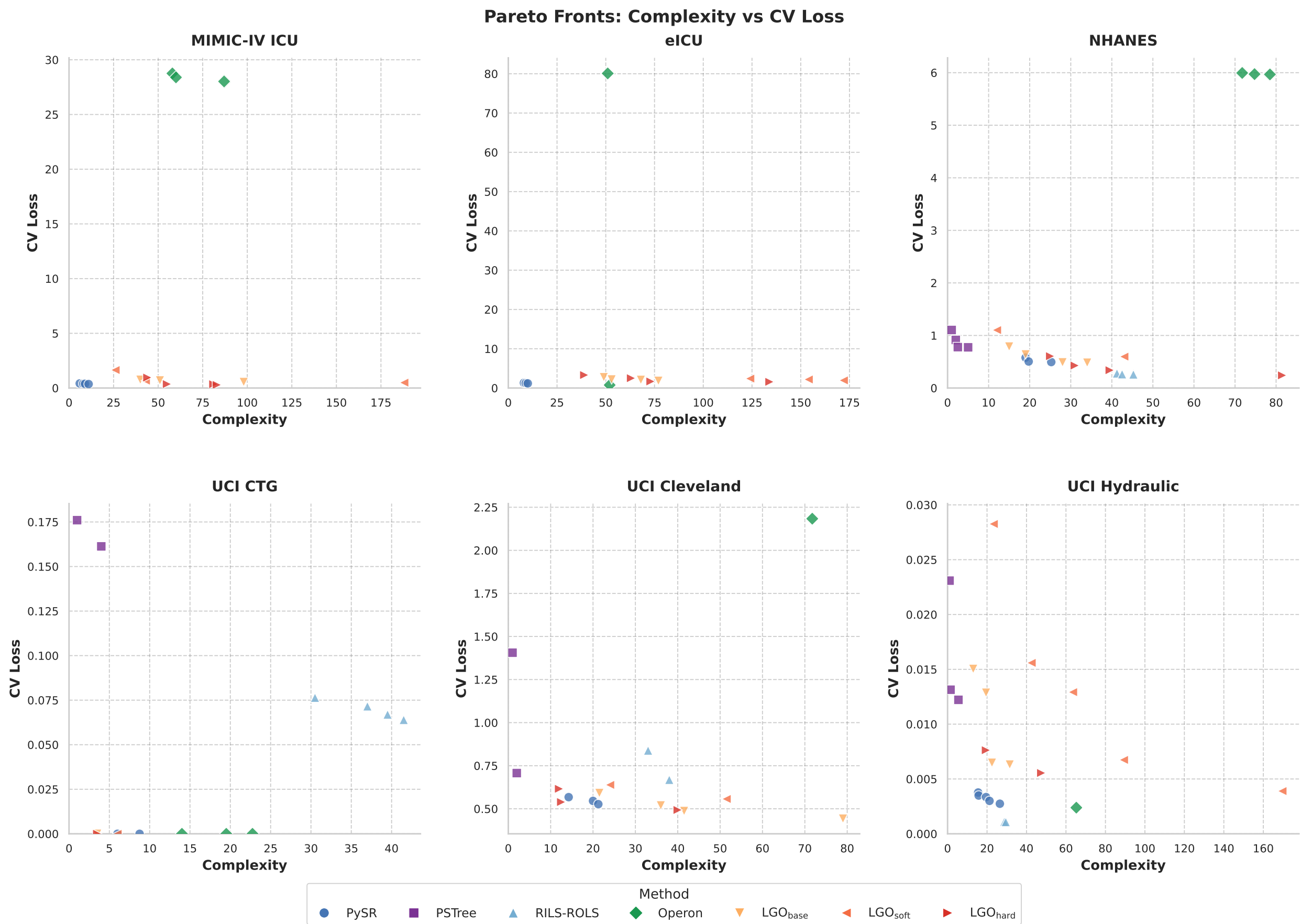

Figure S1: Pareto fronts (CV loss vs. symbolic complexity) for all six datasets. Each panel shows non-dominated candidates aggregated across seeds; colors denote methods. On ICU, eICU, and NHANES, $\mathrm{LGO}_{\mathrm{hard}}$ and $\mathrm{LGO}_{\mathrm{base}}$ appeared in the low-loss region at intermediate complexity, whereas $\mathrm{LGO}_{\mathrm{soft}}$ tended to produce more complex expressions for similar loss and Operon occasionally produced high-loss candidates. On CTG, all engines reached near-zero CV loss and the Pareto fronts largely overlapped. On Cleveland, $\mathrm{LGO}_{\mathrm{hard}}$ achieved lower CV losses at higher complexity while PySR provided lower-complexity alternatives. On Hydraulic, smooth relationships appeared to favour PySR and RILS-ROLS; LGO variants either pruned gates and aligned with these baselines or appeared as higher-complexity points with somewhat higher loss. Related to Figure 2.

baselines; when gates appeared, they focused on a small number of physical variables and could be interpreted as operating-range limits, but they did not appear necessary for predictive accuracy in this system.

Taken together, the UCI benchmarks are consistent with the observations from the clinical datasets: $\mathrm{LGO}_{\mathrm{hard}}$ may be more useful when regime switching and auditability are relevant considerations (as in Cleveland), and it tended to reduce to arithmetic behaviour on near-separable (CTG) or globally smooth (Hydraulic) problems where thresholds may not be the primary mechanism.

S10 PS-Tree hyperparameter sensitivity

The original experiments used a budget-matched PS-Tree configuration (population = 600, generations = 80; evaluation budget = 48,000) to equalise the computational budget across methods. Table S16 compares this configuration with the library default (population = 50, generations = 200; evaluation budget = 10,000), as

Table S13: UCI CTG NSPbin: mean ± std (10 seeds; PS-Tree: 6 seeds[†]). Related to Figure 2.

method	experiment	AUROC↑	AUPRC↑	Brier↓
PySR	base	1.000 ± 0.000	1.000 ± 0.000	0.211 ± 0.000
PS-Tree	base	0.76 ± 0.01	0.51 ± 0.03	0.39 ± 0.02
RILS-ROLS	base	0.517 ± 0.135	0.288 ± 0.110	0.333 ± 0.164
Operon	base	1.000 ± 0.000	1.000 ± 0.000	0.211 ± 0.000
LGO	base	1.000 ± 0.000	1.000 ± 0.000	0.211 ± 0.000
LGO	LGO_{soft}	1.000 ± 0.000	1.000 ± 0.000	0.212 ± 0.002
LGO	LGO_{hard}	1.000 ± 0.000	1.000 ± 0.000	0.211 ± 0.000

[†] PS-Tree: 4 of 10 seeds failed due to prediction-shape mismatch in the classification evaluation pipeline; statistics computed over 6 valid seeds.

Table S14: UCI Heart Cleveland num: mean ± std (10 seeds). Related to Figure 2.

method	experiment	R^2 ↑	RMSE↓	MAE↓
PySR	base	0.416 ± 0.105	0.909 ± 0.085	0.638 ± 0.070
PS-Tree	base	0.01 ± 0.33	1.17 ± 0.12	0.73 ± 0.11
RILS-ROLS	base	0.392 ± 0.127	0.928 ± 0.109	0.645 ± 0.071
Operon	base	0.480 ± 0.107	0.856 ± 0.075	0.600 ± 0.059
LGO	base	0.436 ± 0.112	0.892 ± 0.084	0.574 ± 0.067
LGO	LGO_{soft}	0.397 ± 0.177	0.914 ± 0.108	0.583 ± 0.076
LGO	LGO_{hard}	0.484 ± 0.118	0.852 ± 0.090	0.531 ± 0.061

Table S15: UCI HydraulicSys fault score: mean ± std (10 seeds). Related to Figure 2.

method	experiment	R^2 ↑	RMSE↓	MAE↓
PySR	base	0.738 ± 0.031	0.089 ± 0.004	0.069 ± 0.004
PS-Tree	base	0.41 ± 0.14	0.13 ± 0.02	0.10 ± 0.02
RILS-ROLS	base	0.946 ± 0.007	0.040 ± 0.004	0.028 ± 0.002
Operon*	base	0.822 ± 0.049	0.072 ± 0.010	0.055 ± 0.008
LGO	base	0.609 ± 0.119	0.106 ± 0.017	0.084 ± 0.015
LGO	LGO_{soft}	0.397 ± 0.215	0.131 ± 0.024	0.106 ± 0.021
LGO	LGO_{hard}	0.364 ± 0.248	0.134 ± 0.027	0.107 ± 0.023

Notes: Two seeds excluded due to implausible R^2 values (< -1).

defined in the `PS-Tree` source code (`cluster_gp_sklearn.py`, class `GPRegressor`).

The budget-matched configuration generally outperforms the library default, particularly on eICU and NHANES where the larger population provides greater diversity for the GP component. This confirms that our original experimental design was, if anything, *more favourable* to PS-Tree than the library default would have been. For conservatism, the main-text tables now report PS-Tree results under the library default configuration.

S11 Case studies: single-feature sanity checks (extended plots)

To complement the aggregate threshold audit in the main text (Figure 3), we provide extended single-feature sanity checks for seven feature–dataset pairs: ICU lactate, eICU GCS, NHANES waist circumference and triglycerides, UCI CTG MeanHist, UCI Cleveland resting systolic blood pressure, and UCI Hydraulic System

Table S16: PS-Tree performance under library-default vs. budget-matched hyperparameters (median test R^2 across 10 seeds; CTG uses AUROC, 6 seeds only for default).

Dataset	Default $(50, 200)$	Budget $(600, 80)$	Δ	Budget wins
ICU	0.492	0.500	+0.008	1/10
eICU	0.314	0.535	+0.221	9/10
NHANES	0.242	0.470	+0.228	8/10
CTG	0.755†	—‡	—	—
Cleveland	0.105	0.115	+0.010	3/10
Hydraulic	0.418	0.466	+0.049	5/10

† AUROC, 6/10 seeds (4 seeds failed due to prediction-shape mismatch in the evaluation pipeline).
‡ Budget-match CTG results stored regression R^2, not AUROC; comparison omitted.

PS1 pressure. For each case we plot (A) the feature distribution stratified by the composite-risk (or fault / abnormality) label with guideline and LGO thresholds overlaid, and (B) the ROC curve obtained by using the raw feature as a one-dimensional score, with guideline and LGO operating points marked. These plots illustrate the discriminative capacity of individual features and show where LGO gates fall relative to guideline anchors—whether they appear to adopt more conservative (screening-like) or more stringent (diagnostic-like) operating points.

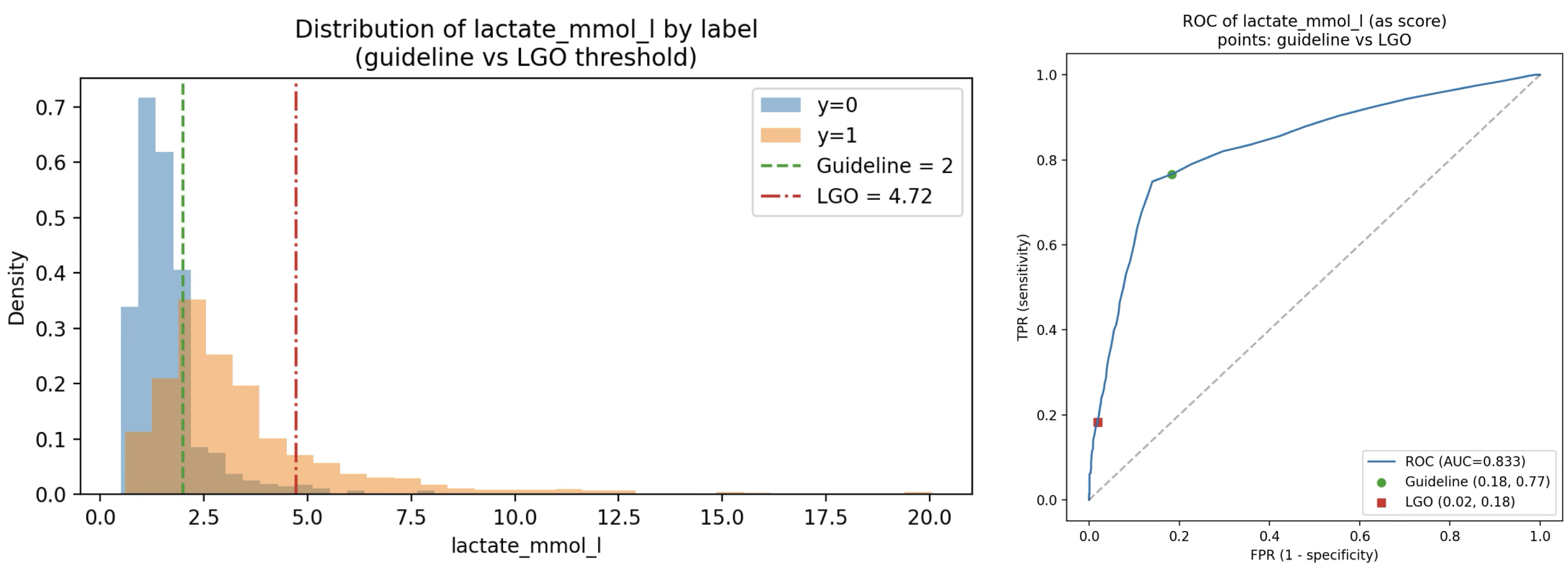

Figure S2: **ICU lactate (composite risk score ≥ 5).** Left panel: distribution of lactate (mmol/L) for low- vs. high-risk patients with vertical lines marking the guideline threshold at 2.0 mmol/L and the $\mathrm{LGO}_{\mathrm{hard}}$ median threshold in the higher-lactate tail. Right panel: ROC curve using lactate as a continuous score (AUC in the high 0.8 range); circles and squares indicate the guideline and LGO operating points, respectively. The guideline cut-off achieved a balanced trade-off between sensitivity and specificity, whereas the LGO gate showed lower sensitivity but higher specificity, which may be consistent with a pattern of flagging only clearly elevated lactate rather than functioning as a screening threshold. Related to Figure 3 and Table 4.

Taken together, these extended plots are consistent with the observations in the main text. On our primary clinical cohorts (ICU and eICU), LGO gates either fell near standard escalation thresholds (MAP, lactate) or shifted toward earlier warning points (GCS, SpO_2). On NHANES, LGO gates for waist circumference and triglycerides appeared close to cardiometabolic screening cutoffs. On CTG and Cleveland, the sanity checks showed both cases of agreement with guideline anchors (MeanHist, `trestbps`) and cases where legacy anchors or coarse encodings may be less applicable (e.g., `FM`, `oldpeak`/`slope`). On Hydraulic, LGO gates fell within engineering reference ranges but showed limited effect on smooth sensor–response ROC curves, which may suggest that threshold primitives are more informative when the underlying mechanism involves regime transitions.

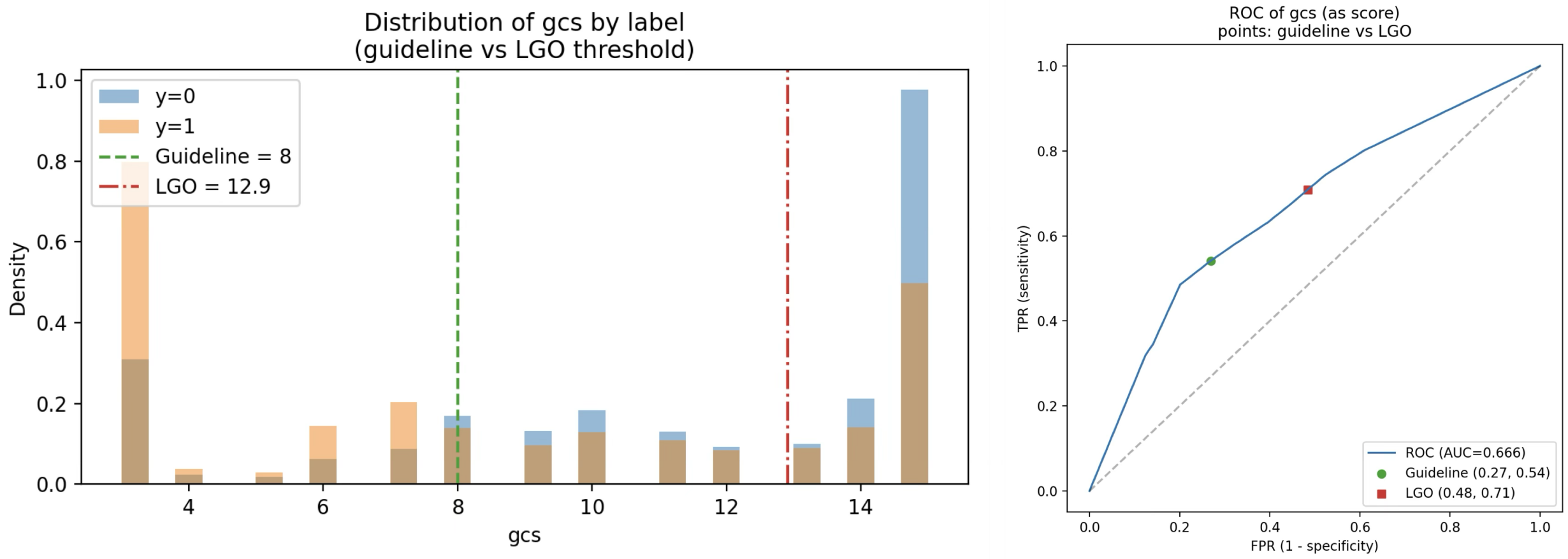

Figure S3: **eICU GCS (composite risk score ≥ 8).** Left panel: distribution of Glasgow Coma Scale (GCS) scores by risk group; vertical lines show the severe-impairment anchor at GCS ≤ 8 and the LGO_{hard} median gate around the low-teens. Right panel: ROC curve for GCS (moderate AUC). The guideline cut-off operated at a relatively specific but less sensitive point, whereas the LGO gate at ~ 13 showed higher sensitivity at the cost of specificity. This pattern may be consistent with an early-warning approach where a drop in consciousness below GCS 13 could trigger attention, even before the patient reaches the classical GCS ≤ 8 boundary. Related to Figure 3 and Table 4.

S12 Extended comparison with clinical scoring and EBM baselines

This section provides the full numerical results for the comparisons between LGO_{hard}, AutoScore, and Explainable Boosting Machines (EBM) on the three primary health datasets. All experiments reuse the same train–test splits as in the main text (ten random seeds). For ICU and eICU we binarize the composite score at pre-specified thresholds (ICU: score ≥ 5; eICU: score ≥ 8); for NHANES we follow the metabolic-syndrome convention (score ≥ 2).

ICU composite risk (binary, score ≥ 5)

Table S*ICU-A* reports mean $\pm$ std over 10 seeds for AUROC, AUPRC, Brier score, F1, accuracy, and training time, comparing LGO_{hard} to AutoScore under a 500k-evaluation search budget. LGO_{hard} achieved somewhat higher AUROC and F1 than AutoScore in these experiments, with a lower Brier score, though at the cost of substantially higher computation time. Table S*ICU-B* provides the analogous comparison between LGO_{hard} and EBM under a lighter 30k-evaluation budget; EBM achieved substantially higher discrimination on this composite endpoint.

Table S17: ICU composite risk (binary, score ≥ 5): LGO_{hard} vs AutoScore (500k evaluations). Mean $\pm$ std over 10 seeds. Related to Figure 5.

Method	AUROC ↑	AUPRC ↑	Brier ↓	F1 ↑	Accuracy ↑	Train time [s] ↓
AutoScore	0.864 ± 0.010	0.904 ± 0.007	0.146 ± 0.005	0.833 ± 0.007	0.784 ± 0.008	0.26 ± 0.08
LGO_{hard}	0.884 ± 0.150	0.905 ± 0.117	0.088 ± 0.074	0.901 ± 0.088	0.878 ± 0.121	151.1 ± 59.8

For robustness, Table S*ICU-C* additionally reports medians and interquartile ranges (IQR) for AUROC, AUPRC, Brier, F1, and accuracy for each method. LGO_{hard} showed a higher AUROC median than AutoScore (approximately 0.94 vs. 0.87), suggesting that the lower mean may have been influenced by a single run with AUROC close to 0.5.

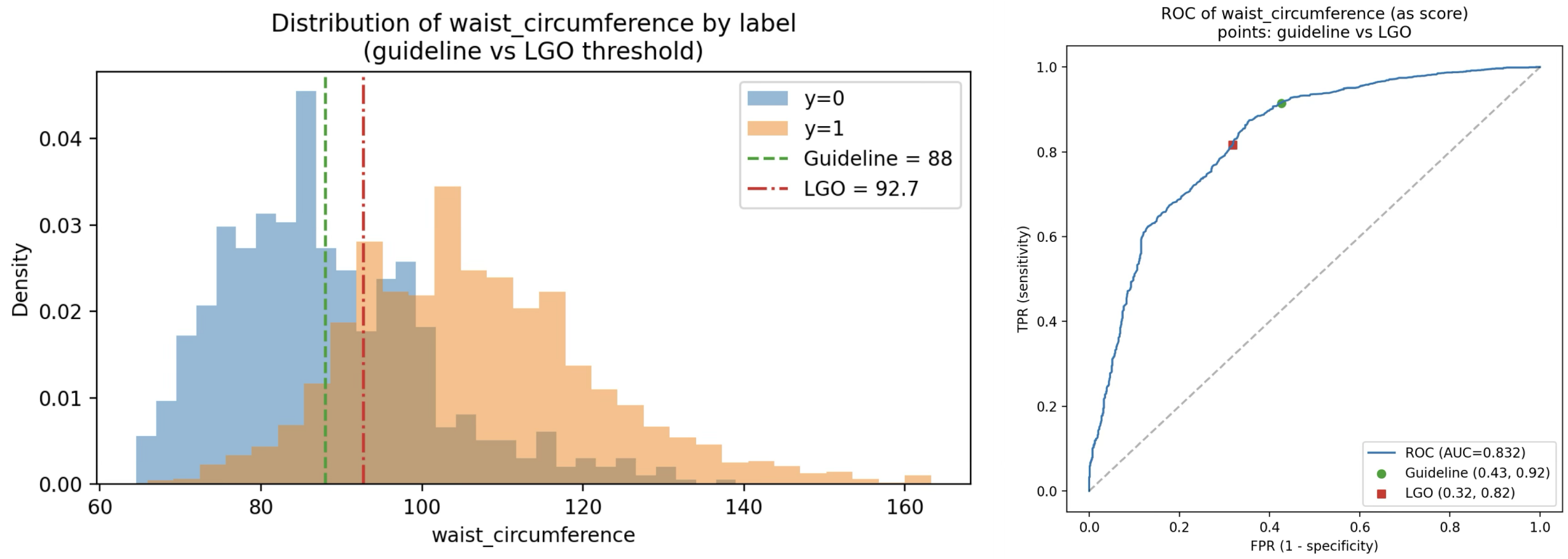

Figure S4: **NHANES waist circumference (metabolic score ≥ 2).** Left panel: distribution of waist circumference (cm) by metabolic-risk status; vertical lines mark the International Diabetes Federation anchor at 88 cm and the LGO_{hard} median threshold in the low-90s. Right panel: ROC curve using waist circumference as a score. Both the anchor and the LGO gate fell near the steep part of the curve, and their operating points showed similar balanced accuracies, suggesting that data-driven and guideline cut-points were relatively close for central adiposity in this dataset. Related to Figure 3 and Table 4.

Table S18: ICU composite risk (binary, score ≥ 5): LGO_{hard} vs EBM (30k evaluations). Mean $\pm$ std over 10 seeds. Related to Figure 6.

Method	AUROC ↑	AUPRC ↑	Brier ↓	F1 ↑	Accuracy ↑	Train time [s] ↓
EBM	0.998 ± 0.001	0.999 ± 0.001	0.010 ± 0.004	0.992 ± 0.003	0.990 ± 0.004	1.12 ± 0.50
LGO_{hard}	0.843 ± 0.131	0.874 ± 0.102	0.198 ± 0.050	0.795 ± 0.065	0.685 ± 0.122	6.28 ± 1.71

eICU composite risk (binary, score ≥ 8)

Table S*eICU* summarizes LGO_{hard} and EBM on the eICU composite-risk endpoint under a 30k-evaluation budget. EBM achieved very high discrimination metrics (AUROC and AUPRC ≈ 1.0; Brier $\approx 6 \times 10^{-4}$; F1 and accuracy ≈ 1.0), while LGO_{hard} achieved AUPRC ≈ 0.90, F1 ≈ 0.95, and accuracy ≈ 0.90, though with more variable AUROC across seeds. As in ICU, these results suggest that LGO_{hard} has lower model capacity than EBM but may still achieve reasonable performance on this task while providing explicit gating structure.

Table S19: eICU composite risk (binary, score ≥ 8): LGO_{hard} vs EBM (30k evaluations). Mean $\pm$ std over 10 seeds. Related to Figure 6.

Method	AUROC ↑	AUPRC ↑	Brier ↓	F1 ↑	Accuracy ↑	Train time [s] ↓
EBM	1.000 ± 0.000	1.000 ± 0.000	0.0007 ± 0.0008	0.9996 ± 0.0004	0.9993 ± 0.0008	3.94 ± 0.78
LGO_{hard}	0.515 ± 0.294	0.897 ± 0.078	0.0921 ± 0.0041	0.945 ± 0.000	0.896 ± 0.001	6.86 ± 0.87

NHANES metabolic risk (binary, score ≥ 2)

Table S*NHANES* reports the same set of metrics for the NHANES metabolic score. EBM achieved high AUROC and low Brier scores, while LGO_{hard} achieved high overall accuracy but substantially lower AUPRC and F1, which may reflect a conservative operating point on this imbalanced outcome. In the main text we therefore focus on NHANES primarily as a case study for threshold recovery and comparison with cardiometabolic anchors, noting that EBM achieved substantially higher predictive performance on this

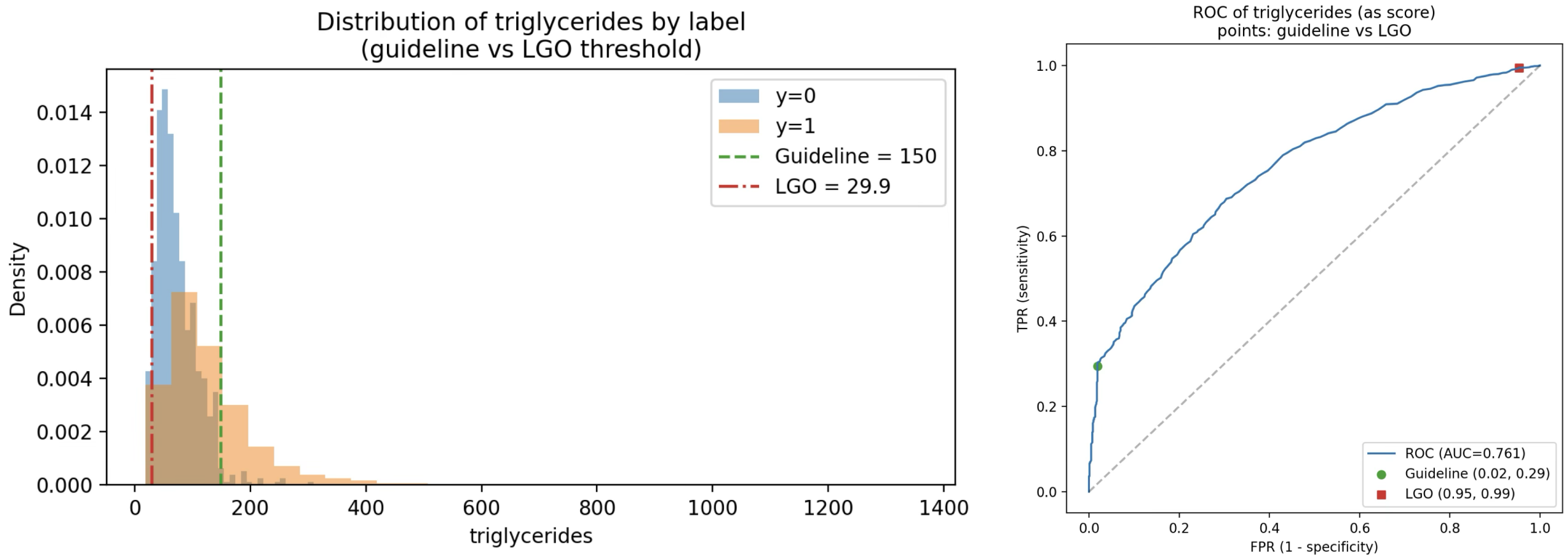

Figure S5: **NHANES triglycerides (metabolic score ≥ 2).** Left panel: distribution of triglycerides (mg/dL) in low- vs. high-risk participants with the 150 mg/dL hypertriglyceridemia anchor and the LGO_{hard} gate overlaid. Right panel: ROC curve (moderate AUC). The guideline cut-off sat slightly to the right of the LGO gate: The LGO gate is substantially lower than the 150 mg/dL anchor in this task, yielding high sensitivity but low specificity. This illustrates that anchor agreement is not guaranteed when the supervised objective favors a different operating point; see the main-text NHANES triglycerides case study. Related to Figure 3 and Table 4.

endpoint.

Table S20: NHANES metabolic risk (binary, score ≥ 2): LGO_{hard} vs EBM (30k evaluations). Mean $\pm$ std over 10 seeds. Related to Figure 6.

Method	AUROC ↑	AUPRC ↑	Brier ↓	F1 ↑	Accuracy ↑	Train time [s] ↓
EBM	0.996 ± 0.003	0.913 ± 0.054	0.0092 ± 0.0030	0.788 ± 0.080	0.988 ± 0.004	0.82 ± 0.37
LGO_{hard}	0.709 ± 0.221	0.134 ± 0.125	0.0299 ± 0.0020	0.011 ± 0.035	0.968 ± 0.003	6.93 ± 1.14

Together, these extended results reinforce the main-text message: LGO_{hard} is competitive with AutoScore on ICU, and retains clinically reasonable performance on ICU and eICU when compared to a strong EBM upper bound, while providing executable, unit-aware thresholds and symbolic formulas rather than point tables or per-feature shape plots.

Baseline coverage.

Table S21: Baseline Selection for Comparative Evaluation. Related to Discussion.

Method Category	Representative Baseline	Included?	Rationale
Clinical Scoring Systems	AutoScore	✓	Point-based risk scores
Additive Models / SET Proxy	EBM (GA^2M)	✓	Shape functions with extractable thresholds
Integer Scoring (MIP-based)	RiskSLIM	×	CPLEX dependency, scalability issues
Symbolic Regression	PySR, Operon, PS-Tree, RILS-ROLS	✓	Direct SR comparisons

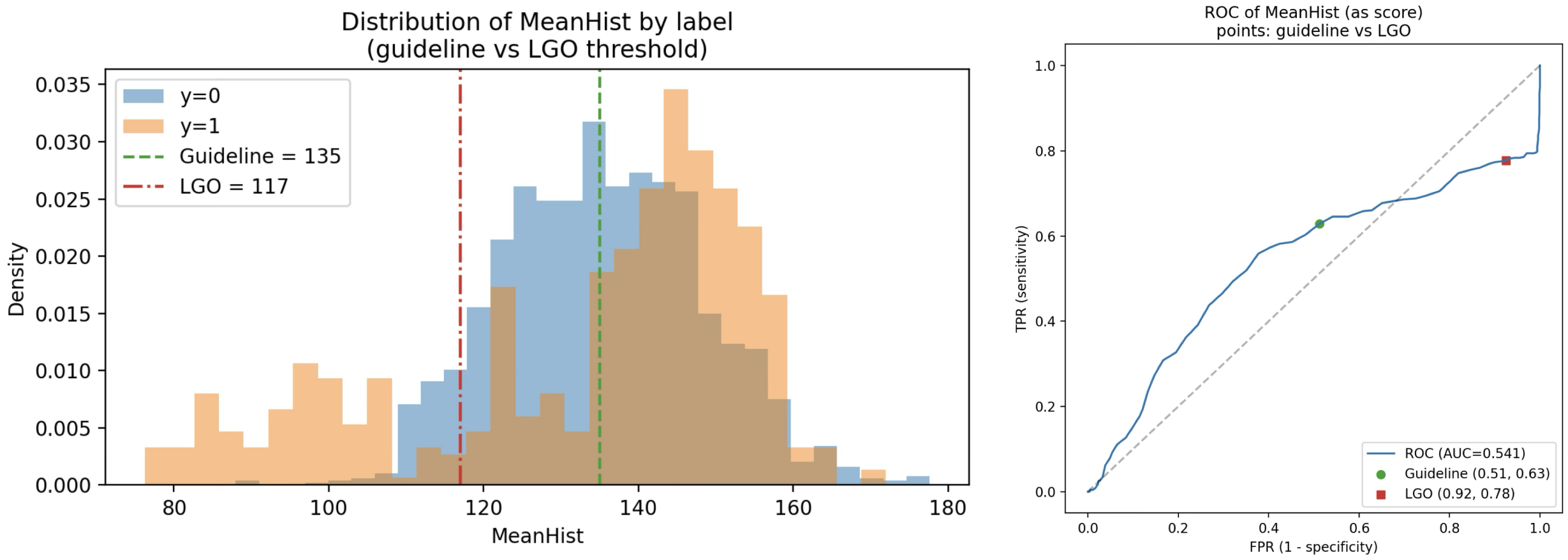

Figure S6: **UCI CTG (NSP binary) – MeanHist.** Left panel: distribution of the histogram mean of fetal heart rate (MeanHist) in normal vs. abnormal traces. The anchor is derived from the curated guideline table (around 135 bpm), with the $\mathrm{LGO}_{\mathrm{hard}}$ gate falling close to this region. Right panel: ROC curve for MeanHist (high AUC, consistent with the near-separable nature of CTG). Both the guideline and LGO operating points fell on the plateau of the ROC, where many thresholds achieved similar performance. In contrast, analyses of features like `FM` (fetal movements; plots not shown) suggest that some anchors inherited from ground-truth tables may be poorly suited to classification (e.g., an anchor at 0), and LGO gates moved toward different cut-points, which may indicate issues with anchor design rather than with the model. Related to Figure 3.

S13 Formal statistical comparison with AutoScore baselines

We compared $\mathrm{LGO}_{\mathrm{hard}}$ to AutoScore on the classification tasks for ICU, eICU and NHANES (score thresholds as in the main text). For each dataset and evaluation budget, we report mean±std AUROC and AUPRC across 10 resampled train–test splits, the difference in AUROC (LGO minus AutoScore), a non-parametric paired Wilcoxon signed-rank test, and effect size (Cohen's d).

Table S22: Performance comparison of $\mathrm{LGO}_{\mathrm{hard}}$ and AutoScore across computational budgets (MIMIC-IV ICU, $n = 10$). Related to Figure 4.

Budget	LGO AUROC	AutoScore AUROC	ΔAUROC [95% CI]	p	Cohen's d	Win
30k	0.927 ± 0.064	0.864 ± 0.009	+0.063 [0.022, 0.098]	0.027^{*}	1.31	7/10
100k	0.910 ± 0.062	0.864 ± 0.009	+0.045 [0.006, 0.083]	0.084	0.96	6/10
200k	0.908 ± 0.062	0.864 ± 0.009	+0.044 [0.005, 0.081]	0.084	0.95	6/10
300k	0.859 ± 0.137	0.864 ± 0.009	−0.006 [−0.100, 0.066]	0.846	−0.06	4/10
500k	0.884 ± 0.142	0.864 ± 0.009	+0.019 [−0.079, 0.091]	0.275	0.18	6/10

∗: $p < 0.05$ (Wilcoxon signed-rank test).
Win: number of seeds where LGO AUROC > AutoScore AUROC.
CI: bootstrap percentile interval (10,000 resamples).

S14 Train–test performance and overfitting

To assess generalization and examine potential overfitting, we computed each model's performance on the training splits and compared it with the held-out test performance. For the regression tasks we report the mean (± standard deviation across methods and seeds) of the difference $\Delta = \mathrm{Train} - \mathrm{Test}$ for each metric. Negative values indicate that the test error is higher than the training error (which may suggest reasonable generalization); positive values indicate the opposite. As summarized in Table S24, these differences were

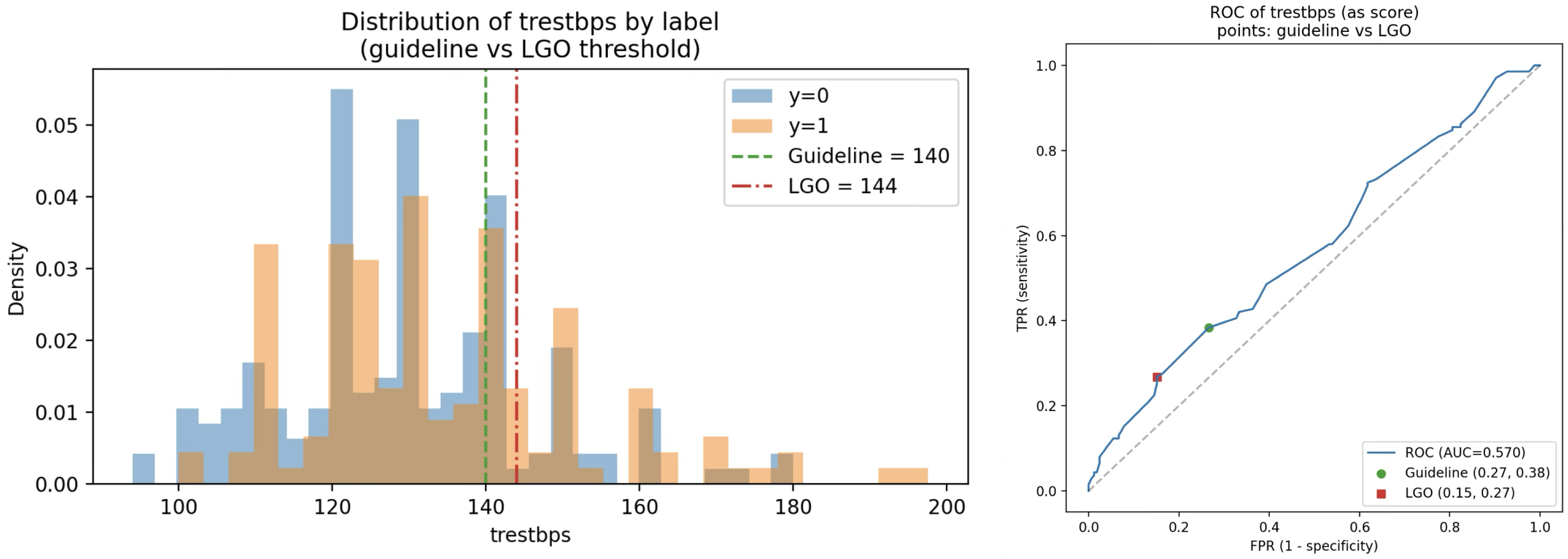

Figure S7: **UCI Cleveland (fault score / risk) – resting systolic blood pressure.** Left panel: distribution of resting systolic blood pressure (`trestbps`, mmHg) by outcome. The anchor around 140 mmHg reflects hypertension guidance; the LGO_{hard} gate typically fell in a similar range, sometimes slightly lower. Right panel: ROC curve for `trestbps` (modest but non-trivial AUC). The LGO operating point showed similar balanced accuracy to the 140 mmHg anchor, while remaining in a comparable range. Sanity checks on categorical encodings such as `cp`, `slope`, or `oldpeak` (results not shown) illustrate how coarse coding constrains the possible thresholds: LGO can only place gates on integer-coded levels, so deviations from anchors there may reflect limitations of the feature representation rather than disagreement with domain knowledge. Related to Figure 3.

Table S23: Performance comparison of LGO_{hard} and AutoScore across computational budgets (eICU, $n = 10$). Related to Figure 4.

Budget	LGO AUROC	AutoScore AUROC	ΔAUROC [95% CI]	p	Cohen's d	Win
30k	0.880 ± 0.078	0.798 ± 0.018	+0.082 [0.030, 0.125]	0.020^{*}	1.37	8/10
100k	0.874 ± 0.066	0.798 ± 0.018	+0.076 [0.028, 0.119]	0.020^{*}	1.49	9/10
200k	0.943 ± 0.019	0.798 ± 0.018	+0.145 [0.130, 0.160]	0.002^{**}	7.38	10/10
300k	0.851 ± 0.130	0.798 ± 0.018	+0.053 [−0.039, 0.117]	0.105	0.54	8/10

∗: $p < 0.05$; ∗∗: $p < 0.01$ (Wilcoxon signed-rank test).
Win: number of seeds where LGO AUROC > AutoScore AUROC.
CI: bootstrap percentile interval (10,000 resamples).

small (typically $|\Delta| \lesssim 0.01$) for ICU, eICU, NHANES and Hydraulic datasets. The UCI Cleveland dataset showed a somewhat larger gap ($\Delta\text{RMSE} \approx 0.08$, $\Delta\text{MAE} \approx 0.05$, $\Delta R^2 \approx 0.15$), though this remained relatively small compared to inter-method variability. For the CTG classification task we report differences in AUROC, AUPRC and Brier score in Table S25; the metrics were similar on train and test splits.

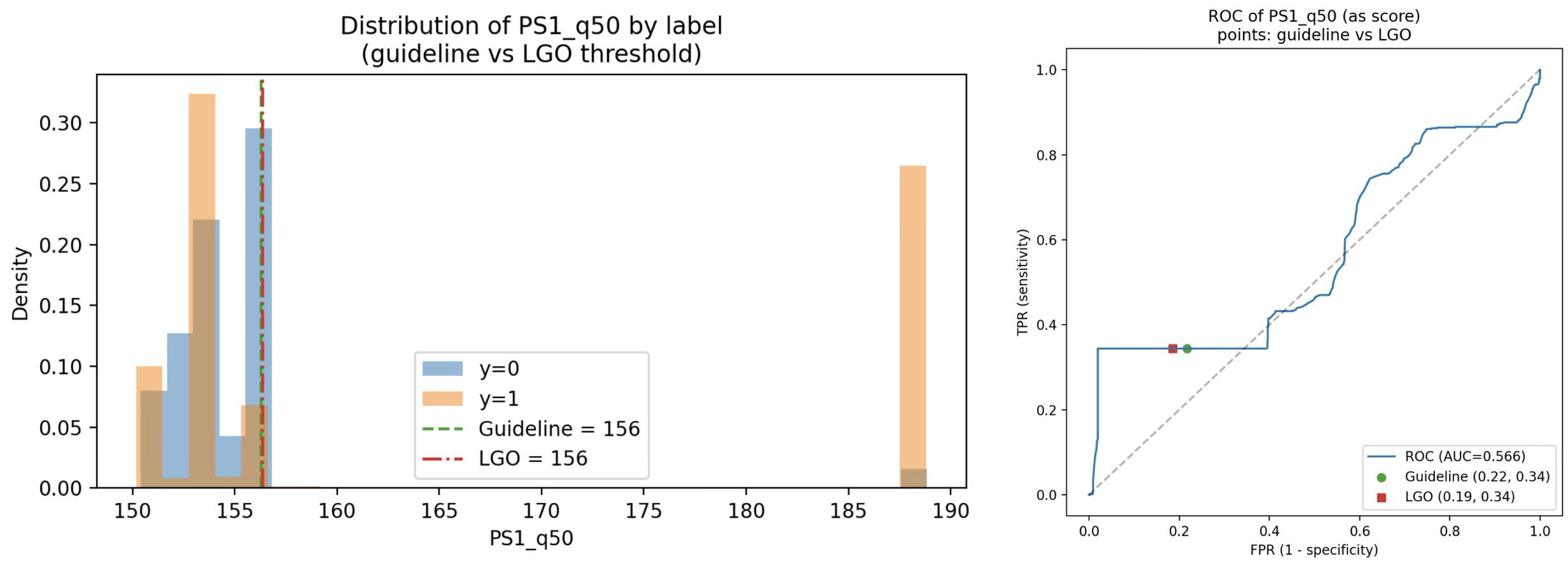

Figure S8: **UCI Hydraulic System (fault score) – PS1 pressure.** Left panel: distribution of the primary pressure sensor statistic (e.g., `PS1_mean` or `PS1_q50`, in bar) across low- vs. high-fault regimes, with the engineering anchor from the guideline table and the $\mathrm{LGO}_{\mathrm{hard}}$ gate overlaid. The gate typically fell close to the suggested operating envelope, suggesting that the learned cut-point may be physically plausible. Right panel: ROC curve for this pressure feature (AUC in the moderate range). The slope is relatively smooth, and moving from the guideline to the LGO gate yielded only small changes in TPR/TNR. This is consistent with our main-text observation that Hydraulic relationships appeared predominantly smooth: LGO thresholds fell near engineering set-points but showed limited additional predictive benefit, which may suggest that gates are more useful on problems where regime switching is present. Related to Figure 3.

Table S24: Train–test performance differences (Δ = Train−Test) on regression datasets. Values are mean ± std across all methods and seeds. Related to Results (Generalization subsection).

Dataset	ΔRMSE	ΔMAE	ΔR^2
ICU	-0.01 ± 0.02	-0.01 ± 0.02	$+0.01 \pm 0.02$
eICU	-0.01 ± 0.02	-0.01 ± 0.02	$+0.00 \pm 0.01$
NHANES	-0.01 ± 0.02	-0.01 ± 0.02	$+0.01 \pm 0.02$
Cleveland	$+0.08 \pm 0.03$	$+0.05 \pm 0.02$	$+0.15 \pm 0.05$
Hydraulic	-0.01 ± 0.01	-0.01 ± 0.01	$+0.00 \pm 0.01$

Table S25: Train–test performance differences on the CTG classification dataset (mean ± std across methods and seeds). The classification metrics match almost exactly on train and test splits, indicating no overfitting. Related to Table S19.

Dataset	ΔAUROC	ΔAUPRC	ΔBrier
CTG	0.00 ± 0.00	0.00 ± 0.00	-0.00 ± 0.00
